# Ultrasonic Interferometric Procedure for Quantifying the Bone–Implant Interface

**DOI:** 10.3390/s23135942

**Published:** 2023-06-26

**Authors:** Jan Lützelberger, Philipp Arneth, Alexander Franck, Klaus Stefan Drese

**Affiliations:** 1 Institute of Sensor and Actuator Technology (ISAT), Coburg University of Applied Sciences and Arts, Am Hofbräuhaus 1b, 96450 Coburg, Germany; philipp.arneth@bestsens.de (P.A.); klaus.drese@hs-coburg.de (K.S.D.); 2 Department of Trauma Surgery and Orthopedics, REGIOMED Clinical Center Coburg, Ketschendorfer Str. 33, 96450 Coburg, Germany; alexander.franck@regiomed-kliniken.de; 3 School of Medicine, University of Split, 21000 Split, Croatia

**Keywords:** quantitative ultrasound, ultrasonic reflection, interferometric measurement, thin layers, film thickness, material characterization, prosthesis loosening, non-invasive technique

## Abstract

The loosening of an artificial joint is a frequent and critical complication in orthopedics and trauma surgery. Due to a lack of accuracy, conventional diagnostic methods such as projection radiography cannot reliably diagnose loosening in its early stages or detect whether it is associated with the formation of a biofilm at the bone–implant interface. In this work, we present a non-invasive ultrasound-based interferometric measurement procedure for quantifying the thickness of the layer between bone and prosthesis as a correlate to loosening. In principle, it also allows for the material characterization of the interface. A well-known analytical model for the superposition of sound waves reflected in a three-layer system was combined with a new method in data processing to be suitable for medical application at the bone–implant interface. By non-linear fitting of the theoretical prediction of the model to the actual shape of the reflected sound waves in the frequency domain, the thickness of the interlayer can be determined and predictions about its physical properties are possible. With respect to determining the layer’s thickness, the presented approach was successfully applied to idealized test systems and a bone–implant system in the range of approx. 200 µm to 2 mm. After further optimization and adaptation, as well as further experimental tests, the procedure offers great potential to significantly improve the diagnosis of prosthesis loosening at an early stage and may also be applicable to detecting the formation of a biofilm.

## 1. Introduction

Due to an increasingly ageing population and a rising demand for a good quality of life, artificial joint replacements, especially hip replacements, have become one of the most common operations in orthopedics and trauma surgery over recent decades and even continue to gain importance [1]. However, in more than 10% of hip replacements, loosening of the implanted prosthesis occurs within the first 15 years after surgical implantation [1,2]. As a result, the prosthesis usually has to be completely replaced in a complex operation which often leads to complications [3,4]. The later the prosthesis loosening is detected, the more difficult the starting situation for a successful and complication-free prosthesis replacement is [4]. In addition to early diagnosis, it is essential to distinguish between purely mechanical (aseptic) loosening and loosening caused by bacterial infection (septic). The latter occurs in approximately 0.4 to 1.5% of cases and requires a much more complex procedure for prosthesis revision than aseptic loosening [5,6].

Thus, there is a great need for medical–technical procedures for the early detection of and differentiation between septic and aseptic implant loosening. To date, however, the diagnosis of loosening in practice is performed almost exclusively by radiological imaging methods, in particular with the aid of classical projection radiography. To distinguish between septic and aseptic loosening, additional laboratory criteria are used and, if necessary, a joint puncture is performed [4,5,7,8].

The disadvantages of two-dimensional radiography, apart from radiation exposure, are poor accuracy and reproducibility, which make reliable diagnosis difficult or even impossible, especially in the early stages of loosening [4,8,9,10,11,12]. An even greater problem is the detection of incipient bacterial accumulation in the form of a biofilm at the bone–implant interface [9]. In this case, a radiological finding can usually only be detected when the patient is already experiencing significant pain [5].

A non-invasive alternative to classical radiography with also significantly higher accuracy and the option of material characterization of the bone–implant interface might be the use of quantitative ultrasound. This refers to the excitation and detection of ultrasound waves in the human body, moving on from the ultrasound imaging established in medicine, with the purpose of obtaining quantitative information about the penetrated tissue [13].

In medicine, research in this field has mainly focused on the ultrasound-based determination of bone thickness, stiffness and porosity for early osteoporosis detection [13,14,15,16]. In principle, however, it is equally possible to obtain quantitative information about the nature of a thin layer, such as that between bone and implant, by using ultrasound [17,18,19,20]. For example, the time- and frequency-related investigation of ultrasound reflected at a thin layer is a typical approach with regard to tribological systems. In this field, it is used to determine the thickness of lubricant films, some of which are less than 100 μm thick, such as those found in ball bearings [21,22,23,24,25,26,27]. However, this approach has not yet been applied to medical problems where rough, non-geometric surfaces as well as very inhomogeneous, highly damping materials with not exactly known probabilities and strong individual deviations are found [28,29,30]. Furthermore, the tribological approach in general relies on a reference measurement with air instead of liquid film, which of course is not possible in the considered medical application.

Therefore, in this work, we combine a well-known analytical model for acoustic reflection at a thin intermediate layer with a new method in data processing to study the bone–implant interface and to develop a corresponding method for characterizing the layer in between without the need for a reference measurement. For this, we exemplarily consider the interface between the femoral bone and the prosthesis stem of cementless hip prostheses. The focus of our experimental studies lay in quantifying the thickness of this boundary layer, with the aim to be able to detect a layer thickness of below 2 mm as this is the lower limit for plain radiography to reliably diagnose aseptic loosening [4,8]. In addition, the method, in principle, also offers the possibility of a physical characterization of the interface to be able to detect the presence of a biofilm.

## 2. Materials and Methods

### 2.1. Analytical Model

**Figure 1:**
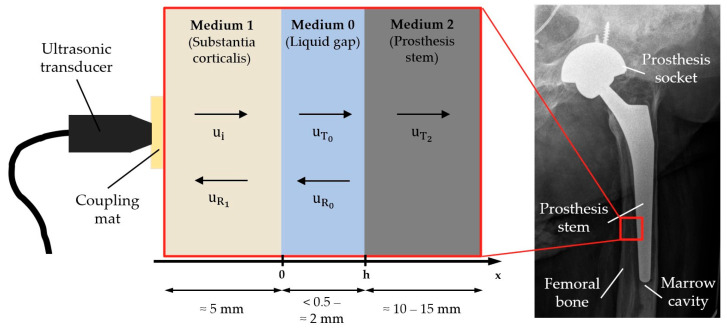
Idealization of the bone–implant interface as a three-layer-system with typical thicknesses and schematic illustration of reflection and transmission.

From a physical perspective, the bone–implant interface to be investigated by ultrasound can be represented by an idealized three-layer system (see Figure 1): on the outside lies the solid substantia corticalis, the cortical bone, followed by a thin liquid demineralized substance as the cleavage medium with thickness h, and finally the prosthesis stem, which is again solid.

Assuming a longitudinal plane wave propagating along the x axis, the deflection u inside a layer can be written as

(1) $$u\left(x,t\right)=\hat{u}e^{i\omega \left(t\;-\frac{x}{c}\right)}$$

with $\hat{u}$ being the wave’s amplitude, ω the circular frequency and c the velocity of the wave.

As Figure 1 shows, an incoming wave $u_{i}$ is transmitted and reflected several times within the three-layer system. Superposing all these transmitted and reflected proportions and omitting the time dependency gives one equation per layer:

(2) $$u_{1}\left(x\right)={\;u}_{i}+u_{R_{1}}=e^{-i\omega \frac{x}{c_{1}}}+R_{1}e^{i\omega \frac{x}{c_{1}}}$$

(3) $$u_{0}\left(x\right)=u_{T_{0}}+u_{R_{0}}={T_{0}\;e}^{-i\omega \frac{x}{c_{0}}}+R_{0}e^{i\omega \frac{x}{c_{0}}}$$

(4) $$u_{2}\left(x\right)=u_{T_{2}}=T_{2}e^{-i\omega \frac{x}{c_{2}}}$$

with the reflection coefficient $R_{k}$ and the transmission coefficient $T_{k}$ of each layer k [19].

Differentiating by x, using general relations for longitudinal waves and applying continuity conditions at the boundaries leads to the following system of linear equations:

(5) $$1+R_{1}=T_{0}+R_{0}$$

(6) $$T_{0}e^{-i\omega \frac{h}{c_{0}}}+R_{0}e^{i\omega \frac{h}{c_{0}}}=T_{2}e^{-i\omega \frac{h}{c_{2}}}$$

(7) $$Z_{1}\left(1\;-R_{1}\right)=Z_{0}\;(T_{0}-R_{0})$$

(8) $$Z_{0}\left(T_{0}e^{-i\omega \frac{h}{c_{0}}}-R_{0}e^{i\omega \frac{h}{c_{0}}}\right)=Z_{2}e^{-i\omega \frac{h}{c_{2}}}$$

where h is the interlayer thickness, $Z_{k}=\rho_{k}c_{k}$ corresponds to the acoustic impedances of each layer and $\rho_{k}$ denotes the density. Solving with respect to $R_{1}=R$ directly results in

(9) $$R=\frac{e^{-i\omega \frac{h}{c_{0}}}\left(Z_{1}+Z_{0}\right)\left(Z_{2}-Z_{0}\right)+e^{i\omega \frac{h}{c_{0}}}(Z_{0}-Z_{1})(Z_{2}+Z_{0})}{e^{-i\omega \frac{h}{c_{0}}}\left(Z_{1}-Z_{0}\right)\left(Z_{2}-Z_{0}\right)-e^{i\omega \frac{h}{c_{0}}}(Z_{0}+Z_{1})(Z_{2}+Z_{0})}$$

for the overall complex reflection coefficient of the three-layer system [24]. From this equation, one can also derive simplified reflection models like Liquid Spring Model [22,23,25,31], Resonance Model [17] or the Time-of-Flight Method [27]. Each of them is only valid for a limited range of thicknesses and frequencies, respectively. As the general model contains all information, the simplifications are not further considered in this work.

**Figure 2:**
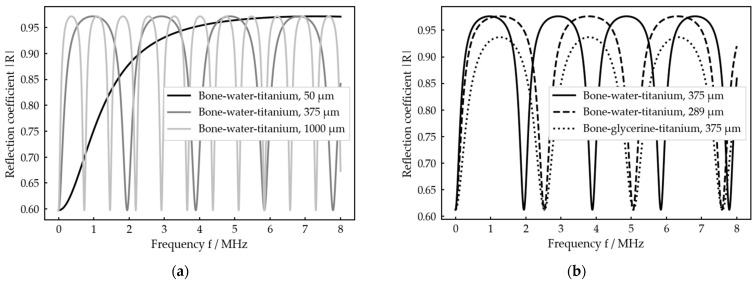
Visualization of the absolute value $\left|R\right|$ of the reflection coefficient over frequency using Equation (9) and the material properties as specified in Table A1 and Table A2: (**a**) variation in thickness of the intermediate layer; (**b**) variation in intermediate medium and thickness.

The reflection coefficient thus results from a combination of the acoustic impedances of the three layers as well as the frequency of the acoustic wave and the ratio of thickness and sound velocity within the intermediate layer. A visualization of the amplitude spectra for different thicknesses of the intermediate layer and different intermediate media is shown in Figure 2.

The spectra show periodic maxima and minima with locations depending on the ratio $h/c_{0}$ and actual shapes depending on the combination of acoustic impedances $Z_{k}$. By changing the thickness h, the whole graph is just stretched and compressed (Figure 2a), while a change in the intermediate medium, which in general goes along with changes in $Z_{0}$ and $c_{0}$, changes the shape of the graph too (Figure 2b). As the ratio $h/c_{0}$ appears in all the complex arguments in Equation (9), it is not possible to distinguish between a change in thickness and a change in the intermediate medium by considering the minima position exclusively. This can only be achieved by taking the shape of the spectral graph into account as it is only influenced by the medium (Figure 2b).

### 2.2. Data Processing

Given the theoretical model described above, information about the thickness and the intermediate medium can be obtained by experimentally determining the spectrum of the reflection coefficient R. With respect to Equation (2) and Figure 1, R is given as

(10) $$R={\left.\frac{u_{R}}{u_{i}}\right|}_{x=0}$$

In the frequency range, we therefore obtain

(11) $$R={\left.\frac{{F\{u}_{R}\}}{F\{u_{i}\}}\right|}_{x=0}$$

with F{u} denoting the Fourier transform of u. As a direct measurement at the border of medium 1 and medium 0 is not possible, usually a reference measurement with air as medium 0 (almost total reflection) is performed to compensate for the influence of the sound path through medium 1 on the acoustic wave. In this case, the reflection coefficient could be directly calculated relative to this reference measurement [21,22,23,24,25,26].

**Figure 3:**
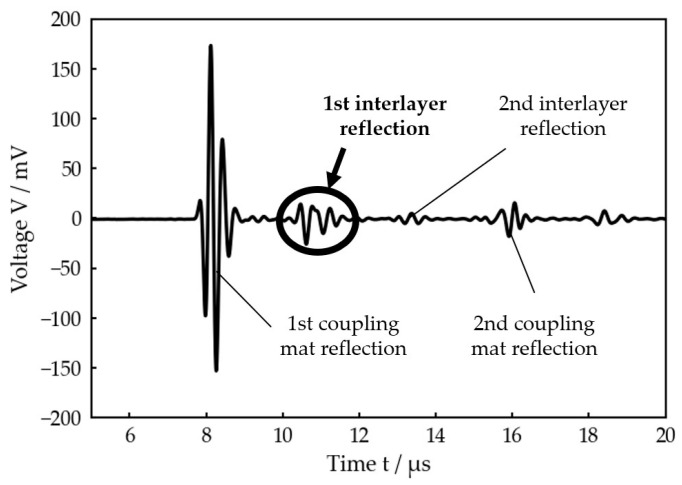
Voltage signal received by the ultrasonic transducer after transmitting a single pulse (planar bone-water–titanium system with calculated interlayer thickness of 221 µm). The first reflection at the interlayer, which is used for the further data processing, is highlighted.

In the considered medical application of an implanted prosthesis, this procedure is not possible, so R cannot be obtained directly. The only relevant quantity which can be experimentally determined in that case is the reflected wave $u_{R}$ on the outside of the bone. Figure 3 exemplarily shows where $u_{R}$ can be found in a typical signal received by an ultrasonic transducer placed on the outside of the bone after transmitting an ultrasonic pulse into a three-layer system. For further data processing, only the first interlayer reflection is included.

Figure 3 also clearly illustrates that a simple Time-of-Flight measurement is not possible in this case because the reflections at the front and back sides of the intermediate layer strongly overlap in the time domain.

In general, $u_{R}$ results from

(12) $$u_{R}\left(t\right)=R\left(f\right)\;g\left(f\right)u_{i}(t)$$

with the incoming wave $u_{i}$ at x=0, the reflection coefficient spectrum $R\left(f\right)$ and the frequency-dependent damping $g\left(f\right)$ in the sound path between x=0 and the measuring point of the transducer. Transferring into the frequency range by performing a Fourier transform gives the experimental spectrum $F\{u_{R}\}$ of the reflected wave

(13) $$F\left\{u_{R}\right\}=R\left(f\right)\;g\left(f\right){F\{u}_{i}\}$$

**Figure 4:**
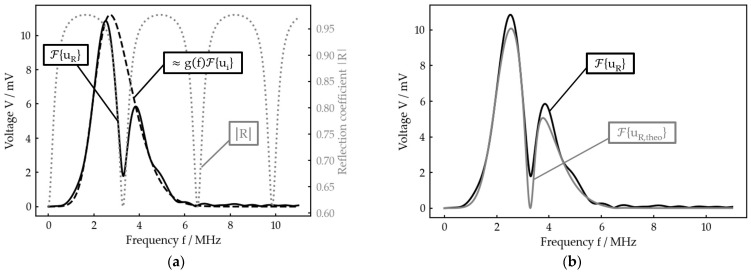
Example for the data processing principle (planar bone–water–titanium system with calculated interlayer thickness of 221 µm): (**a**) theoretical reflection coefficient spectrum $\left|R\right|$, experimental reflection spectrum ${F\{u}_{R}\}$ and reconstructed product $g\left(f\right){F\{u}_{i}\}$ of damping and incoming signal; (**b**) best nonlinear fit for $F\{u_{R,\mathrm{theo}}\}$ to $F{\{u}_{R}\}$.

If one now has a sufficiently broad band incoming signal $u_{i}$, the course of R can be found in $F\{u_{R}\}$, whereas the product $g\left(f\right){F\{u}_{i}\}$ appears as the envelope of $F\{u_{R}\}$ (see Figure 4a). In other words, the reflection coefficient spectrum is windowed by $g\left(f\right){F\{u}_{i}\}$.

Taking this relation into account, a theoretical reflection spectrum $F\{u_{R,\mathrm{theo}}\}$ can be calculated. For this, the product $g\left(f\right){F\{u}_{i}\}$, which can be extracted from $F\{u_{R}\}$, is multiplied with the reflection coefficient $R_{\mathrm{theo}}\left(f\right)$ calculated by Equation (9) for the expected parameter range (e. g. thickness h) with

(14) $$F\{u_{R,\mathrm{theo}}\}=R_{\mathrm{theo}}\left(f\right)\;g\left(f\right){F\{u}_{i}\}$$

Finally, a nonlinear fit of $F\{u_{R,\mathrm{theo}}\}$ to $F\{u_{R}\}$ in dependence of the unknown parameter is performed to determine the very same, for example the current interlayer thickness (Figure 4b).

Note that it is not possible to reconstruct the absolute value of $\left|R\right|$ as, in general, the maximum of $\left|R\right|$ is smaller than 1 (Figure 2a), so there is no frequency at which the incoming wave is totally reflected. Thus, the exact amplitude information of $\left|R\right|$ is lost.

In the experimental part of our work, we focused on determining the layer thickness, assuming the material quantities $Z_{k}$ and $c_{k}$ of each layer to be known. Furthermore, only the amplitude spectrum of R was considered as the phase spectrum does not give any relevant additional information.

For efficient data handling, an algorithm was implemented using the programming language Python. It includes the following processing steps:

1. Determining the experimental spectrum $F\{u_{R}\}$ of the reflected wave by extracting the interlayer reflection $u_{R}$ from the voltage signal received by transducer and oscilloscope and performing a Fourier transformation.
2. Extracting the product $g\left(f\right){F\{u}_{i}\}$ of damping in the sound path of the interlayer reflection and the spectrum of the incoming signal. For this purpose, the envelope of $F\{u_{R}\}$ was modelled using a skew-normal distribution [32,33].
3. Computing the theoretical spectrum $F\{u_{R,\mathrm{theo}}\}$ by calculating $R_{\mathrm{theo}}\left(f\right)$ in the expected range of h, normalizing the interval [0, 1] and multiplying with $g\left(f\right){F\{u}_{i}\}$.
4. Performing a nonlinear least-squares fit of $F\{u_{R,\mathrm{theo}}\}$ to $F\{u_{R}\}$ in the expected range of h. To achieve a more reliable solution, the least-squares optimization is combined with the comparison of the experimental and theoretical positions of the spectral minima. It is only in the case that their positions agree that the solution is accepted as valid. Otherwise, the algorithm does not give back a solution for the thickness.

### 2.3. Measurement Setup

**Figure 5:**
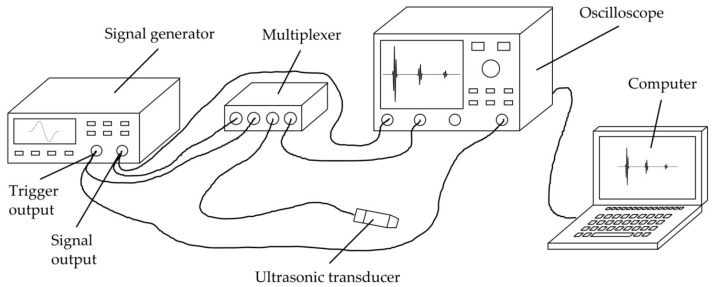
Schematic drawing of the general measurement setup used for all experiments.

The general measurement setup is sketched in Figure 5.

The most crucial component in the setup is the ultrasonic transducer (C384-SU, Evident Corporation, Tokyo, Japan) used for transmitting and receiving the ultrasonic waves, as its signal generation and reception properties are highly relevant for the measurement principle as well as for the specific application. Among other features, the transducer must have an acoustic matching for organic tissue, so that as much energy as possible can be transmitted into the bone. Furthermore, its active surface should be as small as possible, so that a nearly normal incidence of the sound waves within the three-layer system is possible despite the rough surfaces. Moreover, it has to enable a very broad-band transmission, so that the usable frequency window (see Figure 4) is as wide as possible. The unfocused transducer C384-SU meets these requirements by having a central frequency of 3.75 MHz, a peak frequency of 3.45 MHz, a −6 dB-bandwidth ranging from 2.42 MHz to 5.08 MHz, an element size of 6.35 mm and an acoustic matching to water.

A signal generator (33521A, Agilent Technologies, Santa Clara, CA, USA) was used to generate the transmission signal, using a Hanning-windowed single sine wave with 10 V amplitude and a frequency of 3 MHz. While we chose the signal waveform due to its wide and smooth course in frequency domain, the frequency was chosen as a good compromise after performing several preliminary tests with transducers of different frequencies. On the one hand, due to substantial inhomogeneity and porosity, organic materials like bone show a high-frequency-dependent attenuation (see Table A2) which is why low frequencies are required for obtaining a sufficiently strong reflection signal. On the other hand, high frequencies are required for the possibility to determine as small thicknesses as possible (so that minima occur in the experimental reflection spectrum even at very small thicknesses (see Figure 2)).

For acoustic coupling, an Aqualene^®^ coupling mat (Evident Corporation) with a thickness of 6.35 mm was inserted between the transducer and medium 1. It shows a similar sound velocity and acoustic impedance to water or organic tissue. Additionally, the coupling mat was lubricated (Allround Lubricant AL-H, Weicon, Muenster, Germany) on both sides to avoid air inclusions.

A multiplexer (manufactured at ISAT) was used for electrical separation of the signal generator from transducer and oscilloscope during the receiving process for stability reasons. At the oscilloscope (WaveRunner 604Zi, Teledyne LeCroy, Chestnut Ridge, NY, USA), the received signal was averaged (20 measurements each), filtered by a digital low-pass noise filter (−3 dB at 29 MHz) and passed to a computer for further data processing as described in Section 2.2.

## 3. Results

### 3.1. Measurements on Idealized Test Systems

Prior to the test at a bone–implant setup, the developed measurement procedure was validated on two different idealized test systems. For both, water was used as the intermediate medium as its density, sound velocity and sound impedance are near those of the demineralized organic tissue types found in the human body (see Table A1 and Table A2).

**Figure 6:**
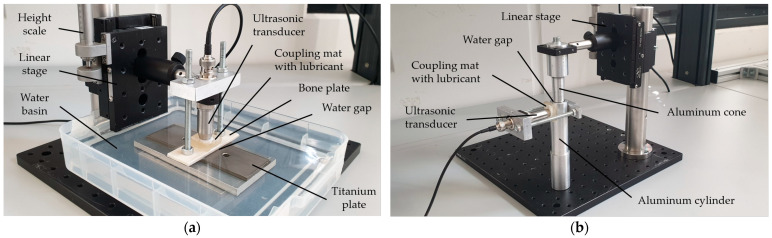
(**a**) Experimental setup for the planar bone–water–titanium system; (**b**) experimental setup for the cylindric aluminum–water–aluminum system.

First a planar bone–water–titanium system (Figure 6a) was used as a configuration with ideal geometric surfaces, but with realistic materials and thicknesses of the layers (see Figure 1). A 4 mm thick plate of leaned and bleached water buffalo bone was used as well as a 6 mm thick plate of a TiAl6V4 alloy, which is frequently used for cement-free hip prosthesis stems [1,31].

Second, a cylindric aluminum–water–aluminum system (Figure 6b) was used to investigate the influence of surfaces being curved in a similar way to the bone–implant interface in the human body. The setup consisted of an aluminum cone and a corresponding hollow cylinder with an outer diameter of 26 mm and a wall thickness of 4.5 mm.

With both setups, a linear stage was used to set a variable thickness of the intermediate layer with a scale precision of 5 µm. While the interlayer thickness was varied in a range of 0.0 to 2.0 mm at the planar setup, it was changed within a range of 0.0 to 1.0 mm at the cylindric setup. For the calculation of R according to Equation (9), the material properties in Table A1 and Table A2 were used for each configuration.

Figure 7 exemplarily shows the results of our measurement procedure, i.e., the optimal fit calculated by the algorithm for each configuration and one adjusted thickness each. For both cases, which represent very different thicknesses, the experimental and theoretical spectra as well as the adjusted and calculated thicknesses show good agreement. In particular, the local minima, which determine the theoretically calculated thickness, fit very well. However, in detail, there are some deviations between experiment and theory which will be discussed later. Figures including the received signal and the best fit for any other adjusted thickness at each setup are given in the Supplementary Materials (Figures S1–S44).

For both setups, all the measurement results, i.e., the thicknesses $h_{\mathrm{cal}}$ calculated by the algorithm, are presented in Figure 8 with respect to the thicknesses $h_{\mathrm{adj}}$ adjusted by the linear stage. A linear fit of $h_{\mathrm{cal}}$ with respect to $h_{\mathrm{adj}}$ was performed for each measurement series and the residuals are depicted against the adjusted thickness.

Overall, Figure 8 demonstrates that $h_{\mathrm{cal}}$ and $h_{\mathrm{adj}}$ are clearly linearly related, showing that our measurement procedure works very well for relatively determining the interlayer thicknesses. Residual values of below 10 µm confirm the high precision of the measurements.

Comparing the linear fits with the ideal relation, one recognizes a slope error of about 3.6% for the planar setup and 2.7% for the cylindric one as well as offsets of −21.6 µm and 7.48 µm, respectively. Both will be discussed later. These errors resulted in a maximum absolute deviation of about 85 µm for $h_{\mathrm{adj}}=2\;\mathrm{mm}$, which is still not problematic with respect to the potential application.

In total, it was possible to determine the interlayer thickness within an interval of 200 µm ≤ $h_{\mathrm{adj}}$ ≤ 2000 µm for the planar bone–water–titanium setup and in the range of 150 µm ≤ $h_{\mathrm{adj}}$ ≤ 600 µm (except $h_{\mathrm{adj}}$ = 500 µm) for the cylindric aluminum–water–aluminum setup.

So, in general, the presented measurement procedure was shown to be appropriate for the measurement task and proved to work precisely even for realistic materials and curved surfaces as required by the potential application.

### 3.2. Measurements on a Bone–Implant System

After the presented measurement procedure was successfully validated at two abstracted test systems, it was applied to a more realistic bone–implant system including the knuckle of a pork (diameter of the marrow cavity about 8 to 10 mm; thickness about 2 mm) and a human hip prosthesis stem consisting of a cobalt–chrome–molybdenum alloy. This should primarily address the question of whether ultrasonic measurements still provide a sufficient signal quality to evaluate implant–bone separation with the presented algorithm.

The experimental setup is shown in Figure 9. In this case, during one measurement series, different interlayer thicknesses were achieved by manually pushing the prosthesis stem inside the bone and then slowly pulling it out while performing several measurements. In total, three measurement series were carried out, including 14 different thicknesses in series 1 and 15 thicknesses in series 2 and 3. For the calculation of R, again the material properties in Table A1 and Table A2 were used.

Due to the limitations of determining the exact separations via a reference method, the measurements were only used to calculate the interlayer thickness by the acoustic procedure. The determined values can be read in Table 1.

For each of the measurements, we provide a figure illustrating the result in the Supplementary Materials (Figures S45–S87). From these values, one can derive that thickness determination with our measurement algorithm is possible within a range of 210 µm ≤ $h_{\mathrm{cal}}$ ≤ 1596 µm. Looking at the resulting thicknesses $h_{\mathrm{cal}}$, their continuously increasing sequence (except for one measurement in series 3) among the same measurement series is consistent with the procedure of pulling the prosthesis stem out of the bone during every series. This further confirms that our measurement approach is successfully applicable to the bone–implant system too.

Figure 10 exemplarily shows the received signal as well as the optimal fit for two different measurements within measurement series 3. With both measurements, the experimental reflection spectrum clearly shows maxima and minima similar to the measurements on the idealized setups, so a thickness calculation by the algorithm was possible, while a time-domain evaluation in general was not.

The second measurement shown (Figure 10b) was measured but with the prosthesis stem pulled further out of the bone than with the first one (Figure 10a). In agreement with that, the calculated thickness is larger for the second one compared to the first one. Looking at the received signals, this is also confirmed, as in Figure 10b the reflection on the back side of the intermediate layer was already separated from that on the front side, while for Figure 10a it was not yet.

## 4. Discussion

The presented measurement procedure proved to be able to successfully determine interlayer thicknesses among all experimental setups, especially including bone measurements.

At two idealized test systems, a planar bone–water–titanium setup and a cylindric aluminum–water–aluminum setup, thickness determination was possible within a range of 200 µm ≤ $h_{\mathrm{adj}}$ ≤ 2000 µm and 150 µm ≤ $h_{\mathrm{adj}}$ ≤ 600 µm, respectively. Among all measurements, there was good agreement between the adjusted and computed interlayer thicknesses. Moreover, the theoretical and experimental reflection spectra showed good agreement too, so the theoretical model combined with our data processing approach generally simulates the real reflection process very well.

Furthermore, we performed investigations on a bone–implant system. It represented more realistic conditions including inhomogeneous and porous bone with its rough surface as well as a real hip prosthesis stem with its special surface shape. Nevertheless, we showed that the frequency domain signal contained sufficient information for determining interlayer thicknesses within a range of 210 µm ≤ $h_{\mathrm{cal}}$ ≤ 1596 µm, although under much more challenging circumstances.

Beside these promising results, one nevertheless recognizes some deviations between the thicknesses calculated by the algorithm and the ones adjusted by the linear stage (for the idealized test setups), but also between experimental and theoretical reflection spectra. Moreover, the whole thickness range up to about 2 mm could not be covered in any case. These three main aspects are considered in the following discussion.

### 4.1. Deviations between Thickesses Calculated by the Algorithm and Those Set by the Linear Stage

Looking at Figure 8, there are both slope and offset errors leading to deviations between the thickness determined by the algorithm and the thickness adjusted by the linear stage.

The slope errors of the regression lines represent a constant relative error of the thickness determined by the algorithm with respect to the adjusted thickness. This can be explained by the fact that thickness h and sound velocity $c_{0}$ are non-separable in the calculation of the theoretical reflection coefficient (Equation (9), Section 2.2). Consequently, an inaccuracy of the value of $c_{0}$ used for the calculation directly causes a relative error of h with $\delta h/h=\delta c_{0}/c_{0}$.

The exact value of $c_{0}$ is dependent on both water temperature and salinity [34]. As the goal of the investigations on the idealized systems was not to perform accurate absolute measurements but rather to validate that the algorithm works, we did not determine the actual value of $c_{0}$, but used a calculated value of 1460 m/s (Table A2). This corresponds to about 13 °C assuming the water is pure [35]. Correcting the relative error of about 3.6% occurring for the planar bone–water–titanium system would require a sound speed value of 1512 m/s which corresponds to about 28 °C, assuming a salinity of 5‰ [34]. As either the temperature or the salinity was monitored during the experiments, it is very likely that the resulting inaccuracy in $c_{0}$ does sufficiently explain the relative error in h.

Considering the perspective application and the range of sound velocities for demineralized organic tissue (Table A2), they show a spread of about 8%, resulting in a potential thickness error of 8%. For a thickness of 2 mm, this would give a worst-case absolute deviation of about 164 µm, which is not really critical for medical applications as this is still within the typical roughness of the surfaces involved.

For the cylindric aluminum–water–aluminum system, the relative error may also be due to manufacturing inaccuracies or the specific construction of the setup.

The offsets of about −21.6 µm and 7.48 µm, respectively, are due to the manual determination of the zero point of the adjusted thickness for the test systems, which was performed optically only.

Correcting the thickness deviations by subtracting the linear proportions leads to residuals of below 10 µm, which shows the potential accuracy that could be achieved by the algorithm when $c_{0}$ is exactly known.

### 4.2. Limitations in Thickness Determination by the Algorithm

For all the investigated configurations, the minimal measured thickness was about 200 µm due to the limited frequency window in which spectral minima can occur, while for the setups with realistic materials, the surface roughness also plays a role in the range of such small thicknesses. However, this is not critical, as the medical application does not demand such small thicknesses.

The measurement principle itself does not set an upper limit for thicknesses to be determined, but the experimental setups do. One important aspect is the overlapping of the reflection at the back side of the interlayer with the second reflection within medium 1 itself, causing unwanted interferences that occur in the spectrum. If medium 1 is relatively small, this could already occur at small thicknesses, like it did at the cylindric aluminum–water–aluminum system and therefore prevented thickness determination above about 600 µm. Fortunately, in the real application case, this is less likely, as sound velocities in bone are lower than in aluminum and bone tissue shows a much higher attenuation, so the second reflection within the bone might even have negligible amplitudes. Figure 3 suggests this.

### 4.3. Deviations between Theoretical and Experimental Reflection Spectra

**Figure 7:**
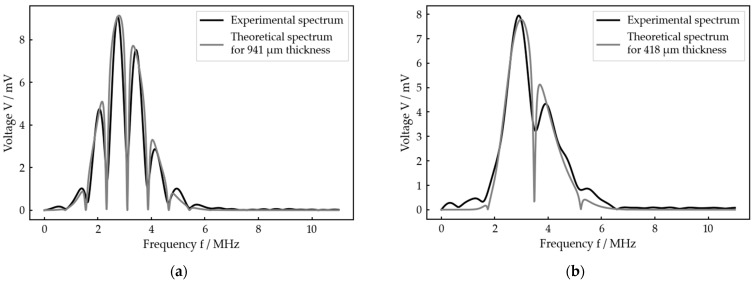
Exemplary result for each of the two idealized test systems: (**a**) best fit for an adjusted interlayer thickness of 1000 µm at the planar bone–water–titanium system; (**b**) best fit for an adjusted interlayer thickness of 400 µm at the cylindric aluminum–water–aluminum system.

**Figure 8:**
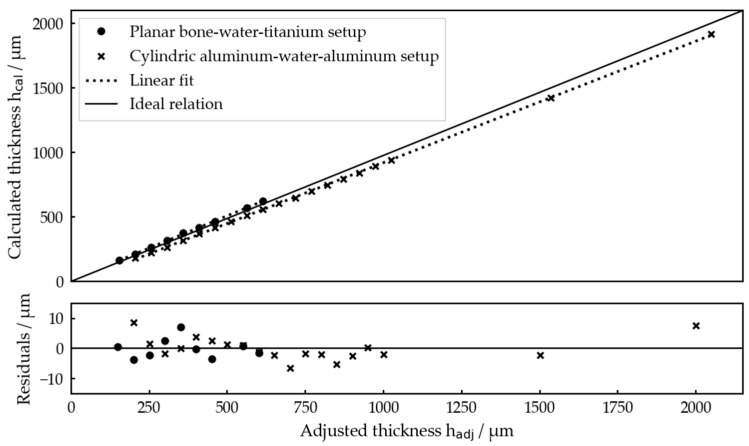
Interlayer thicknesses determined by the algorithm for both idealized test systems for each thickness set by the linear stages and residuals of the linear fits. The linear fits gave $h_{\mathrm{cal}}=0.964\;h_{\mathrm{adj}}-21.6\;µm$ for the planar bone–water–titanium system and $h_{\mathrm{cal}}=1.027\;h_{\mathrm{adj}}+7.48\;µm$ for the cylindric aluminum–water–aluminum system.

**Figure 9:**
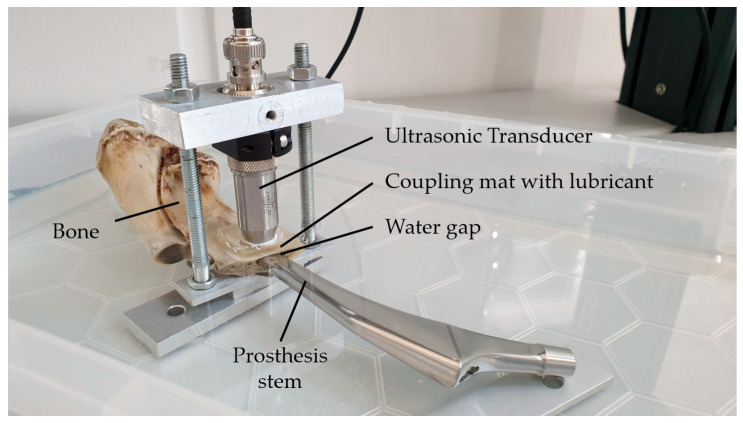
Experimental setup for the bone–implant system.

**Figure 10:**
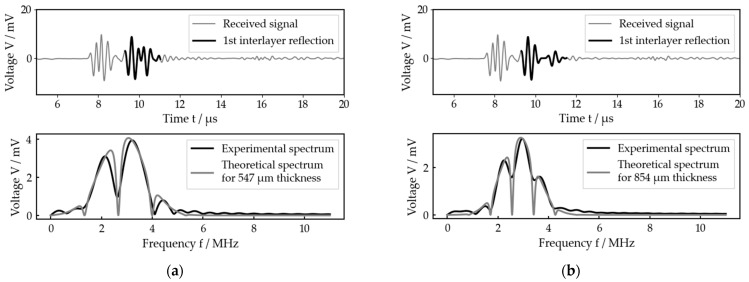
Two exemplary measurements for the bone–implant system (both from measurement series 3), each with received signal and best fit: (**a**) measurement 10 (calculated interlayer thickness of 547 µm, prosthesis stem further inside the bone); (**b**) measurement 14 (calculated interlayer thickness of 854 µm, prosthesis stem further outside the bone).

For all the fit results which were presented here (Figure 4, Figure 7 and Figure 10), the theoretical spectra do represent the principal course of the experimental reflection spectra, especially the minima location, but they do not perfectly fit together. Up to a certain level, the inhomogeneity of the materials as well as scattering at rough surfaces (especially for the bone) might explain the deviations, especially small additional ripples in the spectra which sometimes caused the algorithm to fail with the thickness determination because the minima in the theoretical and the experimental spectra did not fit to each other.

**Figure 11:**
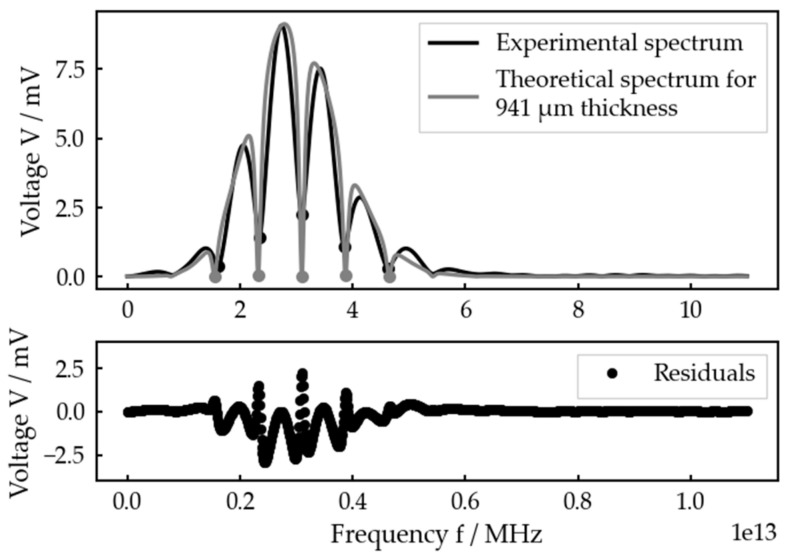
Best fit for an adjusted interlayer thickness of 1000 µm at the planar bone–water–titanium system and residuals.

But taking a closer look at a measurement with a very smooth experimental spectrum (Figure 11), one can see that there seems to be a systematic error in the theoretical spectrum in contrast to the experimental one. Plotting a quantile–quantile plot of the residuals against normal distributed data confirms that the deviations cannot be explained by random noise or any other random error (see Figure S88).

In consequence, this means that the model used for building the theoretical spectrum does not capture every relevant dependency of the experimental spectrum.

One the one hand, the attenuation within the interlayer was not considered. This could have led to an additional exponential factor in Equation (1) and therefore finally change the resulting reflection coefficient spectrum, although the expected changes would be small due to a very small attenuation coefficient of water (Table A2).

On the other hand, dissipation within the interlayer was neglected too. In contrast to the model used, the transmitted acoustic waves are not plain, but their geometry strongly depends on the transducer characteristics. Some of the energy transferred into the interlayer therefore is laterally distributed and the reflected wave has less energy than the model predicts. This phenomenon gains importance with thicker intermediate layers.

Furthermore, the analytical model includes all the multiple reflections while our algorithm only takes the first one. Finally, the material parameters used for calculating the reflection coefficient, especially those of organic materials (Table A1), vary in a wide range and therefore could lead to an error in the shape of the spectral maxima too.

The magnitude of influence that all these factors have on the theoretical reflection spectrum was not investigated during our studies and is left to be clarified in future work.

## 5. Conclusions

In this paper, a new measurement procedure for quantification of the bone–implant interface in prostheses was introduced. Based on a well-known acoustic reflection model, the theoretical spectrum of an ultrasonic pulse reflected at a thin, liquid intermediate layer between two solid materials was computed and a nonlinear fit in dependency of the required parameter to the experimentally measured spectrum was performed. A reference measurement was not needed to run the algorithm.

With respect to thickness determination (assuming known material parameters), the approach was successfully validated at two idealized test systems in the range of about 200 µm to 2 mm. Finally, the technique was applied to a bone–implant system, where the algorithm was able to determine interlayer thicknesses in a range of about 200 µm to 1.6 mm and was shown to also be applicable for inhomogeneous, porous materials with rough surfaces as they occur in the potential application. Deviations that appeared between adjusted and calculated thicknesses as well as between theoretical and experimental spectra could be generally explained.

The possible next steps include validation at a realistic bone–implant system (e.g., fresh bones with skin, fat and muscle tissue) including a comparative measurement for evaluating the accuracy of the results.

Although the algorithm theoretically offers the possibility for the material characterization of the interlayer, i.e., it could be potentially applied to detect the formation of a biofilm, this must be further investigated as well as experimentally validated.

Finally, the analytical model and the data processing should be optimized to minimize the systematic error of the theoretical spectra. For example, a recursive algorithm can be used for modelling the reflections in the time domain, so inferred influences like damping and dissipation could be included. Additionally, this would offer the possibility to only include the first interlayer reflection in the calculation.

## Figures and Tables

**Table 1:** Interlayer thicknesses calculated for each measurement in at the bone–implant setup. If no thickness could be calculated, this is annotated with a dash.

$\mathrm{Thicknesses}\;h_{\mathrm{cal}}$ in Series 1/μm	$\mathrm{Thicknesses}\;h_{\mathrm{cal}}$ in Series 2/μm	$\mathrm{Thicknesses}\;h_{\mathrm{cal}}$ in Series 3/μm
−, −, −, −, −, −, −, −, 210, 556, 625, 935, 1255, 1596	−, −, −, −, −, −, −, −, 311, 393, 514, 573, 852, 1016, 1479	−, −, −, 523, −, −, 405, 443, 453, 547, 656, 728, 797, 854, 1510

## Data Availability

The data presented in this study are available on request from the corresponding author.

## Supplementary Materials

The following supporting information can be downloaded at https://www.mdpi.com/article/10.3390/s23135942/s1. The material contains figures with the received signal and best fit for every measurement on each setup. Figures S1–S23 refer to the planar bone–water–titanium system, Figures S24–S44 to the cylindric aluminum–water–aluminum system and Figures S45–S87 to the bone–implant system. For the two idealized setups, the adjusted thickness is given in each figure caption, while for the bone–implant system S denotes the measurement series and M indicates the measurement number. Figure S88 shows the quantile–quantile plot of the residuals for the planar bone–water–titanium setup with an adjusted thickness of 1000 µm.

## Appendix A

**Table A1:** Relevant mechanical properties of selected materials/media. For calculations involving the properties of bone, the means of the provided intervals were used.

Material/Medium	Density ρ kg/m^3^	Young’s Modulus E GPa	Poisson Number ν	Compressibility χ 1/GPa
Aluminum [36]	2702	70	0.34	−
Titanium [36]	4150	110	0.35	−
Cobalt–chrome–molybdenum [37]	8290	241	0.3	−
Bone (Substantia corticalis) [38]	1800 … 1990	13 … 18.2	0.22 … 0.42	−
Aqualene^®^ [39]	920	1.58	0.337	−
Water (20 °C) [36]	998	−	−	0.47
Glycerin [36]	1261	−	−	0.22
Skin tissue [40]	1150	−	−	−
Fat tissue [40]	950	−	−	−
Muscle tissue [40]	1065	−	−	−

**Table A2:** Relevant acoustic properties of selected materials/media. Sound velocity was calculated via $c=\sqrt{E\;(1\;-\;\nu )/(\rho \;\left(1\;-\;\nu \;-\;2\nu^{2}\right))}$ or $c=\sqrt{1/(\chi \;\rho )}$, respectively, for each material (except skin, fat and muscle tissue) with the characteristics in Table A1. For bone, the mean of the given parameter ranges was chosen. Acoustic impedance was calculated through Z=ρ c for all materials.

Material/Medium	Longitudinal Sound Velocity c m/s	Acoustic Impedance Z MNs/m^3^	Attenuation Coefficient α dB/(cm MHz)
Aluminum	6315	17.1	0.014 … 0.15 [41]
Titanium (alloy)	6522	27.1	0.15 … 0.70 [41]
Cobalt–chrome–molybdenum	6256	51.9	−
Bone (Substantia corticalis)	3432	6.50	1 … 10 [38]
Aqualene^®^	1616	1.49	2.8 dB/cm at 5 MHz
Water (20 °C)	1460	1.46	0.002 [42]
Glycerin	1899	2.39	−
Skin tissue	1730 [40]	1.99	−
Fat tissue	1460 [42]	1.39	0.8 [42]
Muscle tissue	1580 [42]	1.68	1.2 [42]
