# Supplementary figures and images for: Ultrasonic Interferometric Procedure for Quantifying the Bone–Implant Interface
Source: Sensors (Basel). 2023 Jun 26;23(13):5942. doi: 10.3390/s23135942 (PMC10346919; doi:10.3390/s23135942)

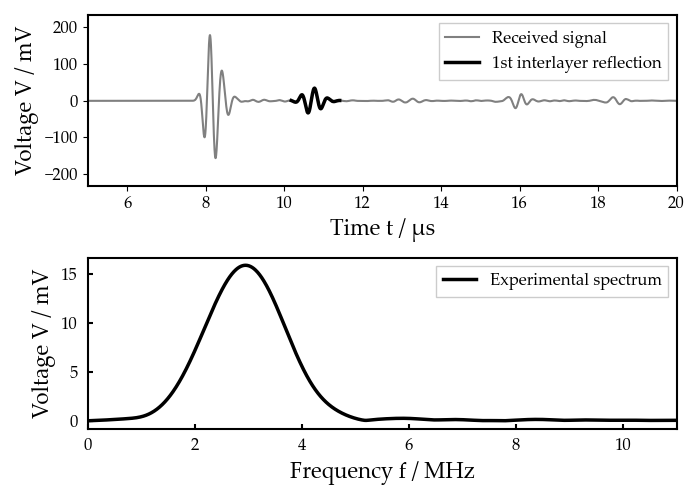

Supplement: Supplementary file 1 [file sensors-23-05942-s001.zip › Figure_S1_Planar_Bone-Water-Titanium-Setup_0000um.png]

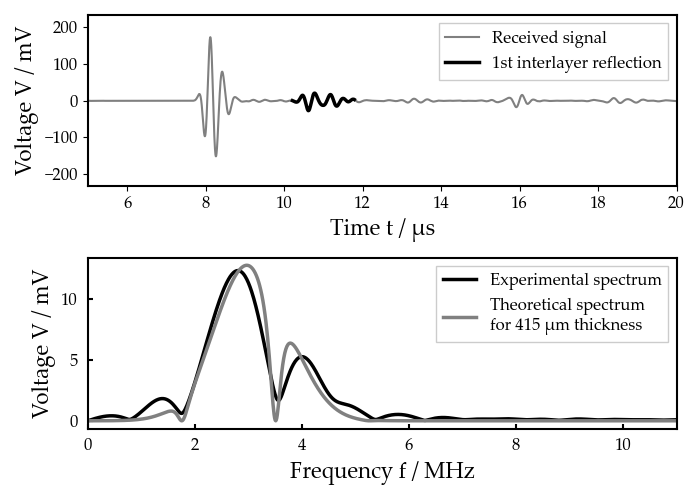

Supplement: Supplementary file 1 [file sensors-23-05942-s001.zip › Figure_S10_Planar_Bone-Water-Titanium-Setup_0450um.png]

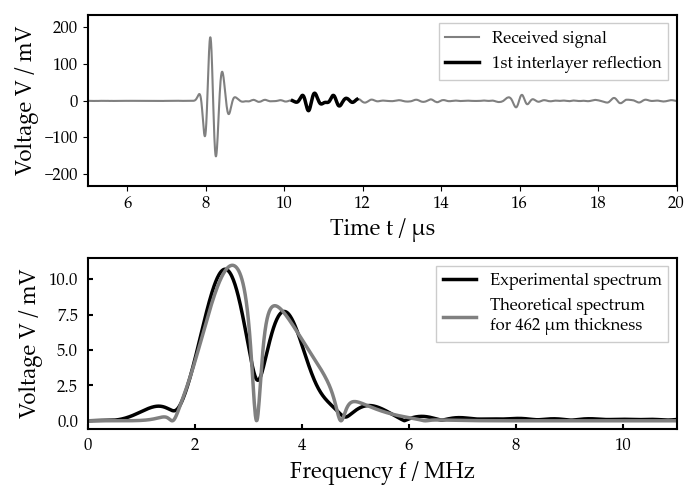

Supplement: Supplementary file 1 [file sensors-23-05942-s001.zip › Figure_S11_Planar_Bone-Water-Titanium-Setup_0500um.png]

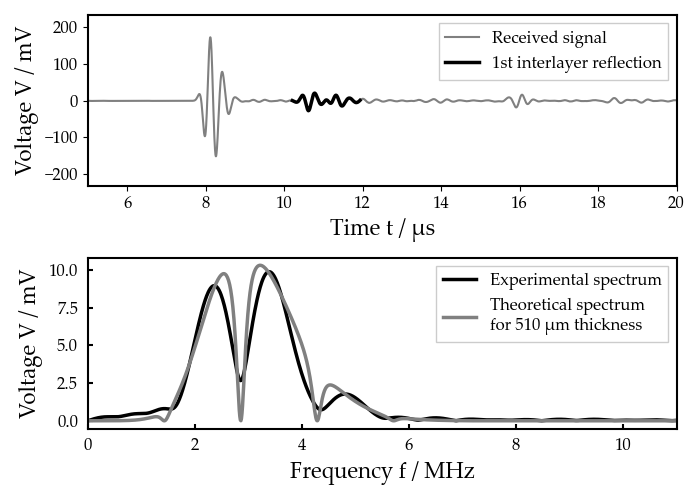

Supplement: Supplementary file 1 [file sensors-23-05942-s001.zip › Figure_S12_Planar_Bone-Water-Titanium-Setup_0550um.png]

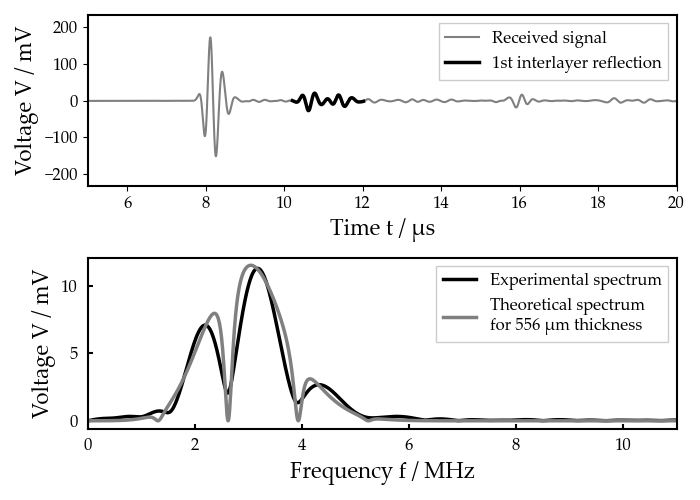

Supplement: Supplementary file 1 [file sensors-23-05942-s001.zip › Figure_S13_Planar_Bone-Water-Titanium-Setup_0600um.png]

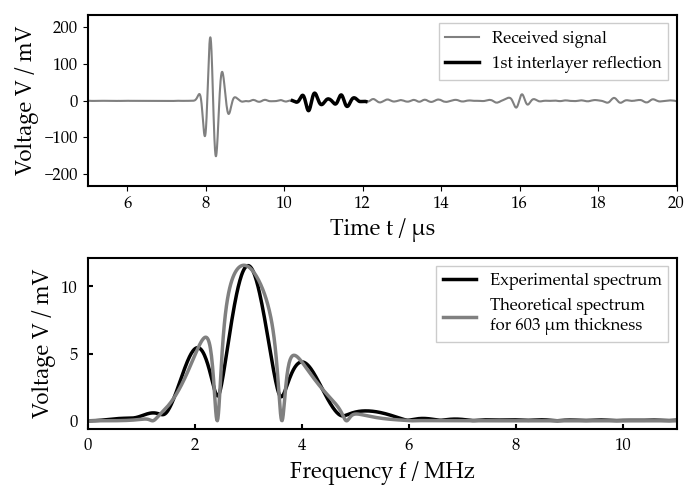

Supplement: Supplementary file 1 [file sensors-23-05942-s001.zip › Figure_S14_Planar_Bone-Water-Titanium-Setup_0650um.png]

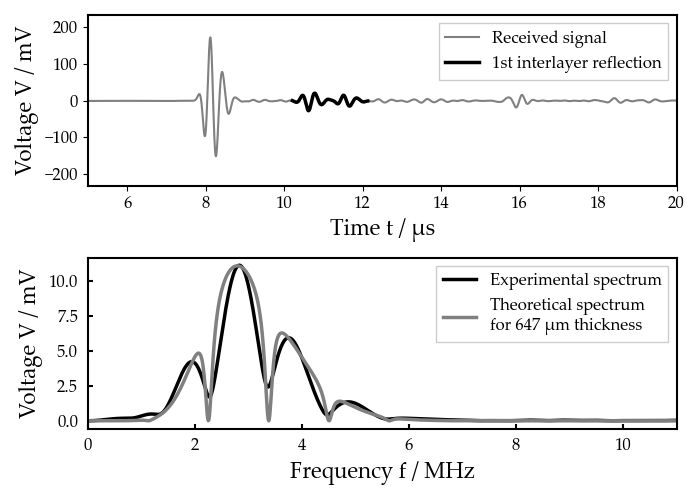

Supplement: Supplementary file 1 [file sensors-23-05942-s001.zip › Figure_S15_Planar_Bone-Water-Titanium-Setup_0700um.png]

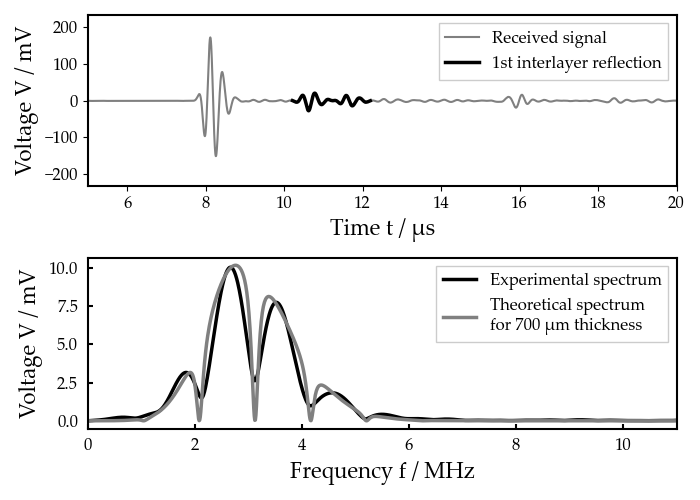

Supplement: Supplementary file 1 [file sensors-23-05942-s001.zip › Figure_S16_Planar_Bone-Water-Titanium-Setup_0750um.png]

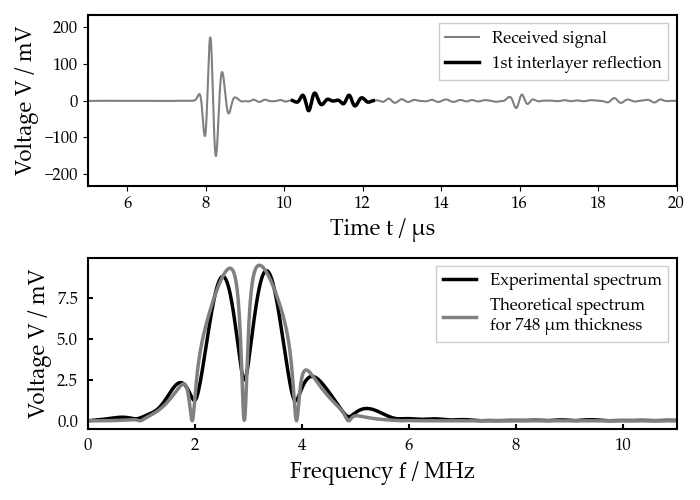

Supplement: Supplementary file 1 [file sensors-23-05942-s001.zip › Figure_S17_Planar_Bone-Water-Titanium-Setup_0800um.png]

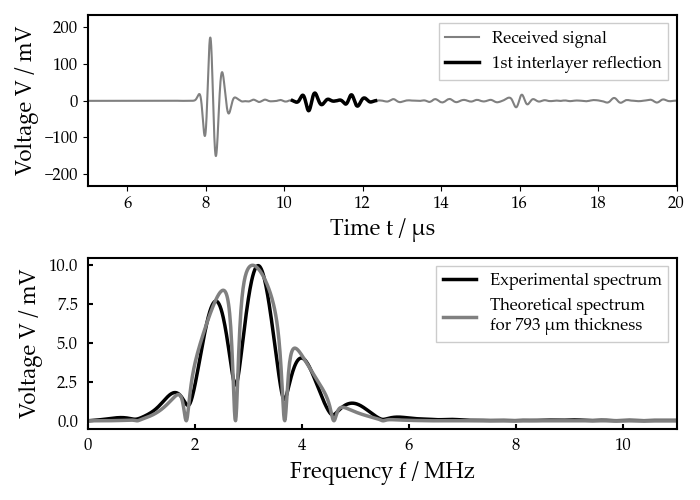

Supplement: Supplementary file 1 [file sensors-23-05942-s001.zip › Figure_S18_Planar_Bone-Water-Titanium-Setup_0850um.png]

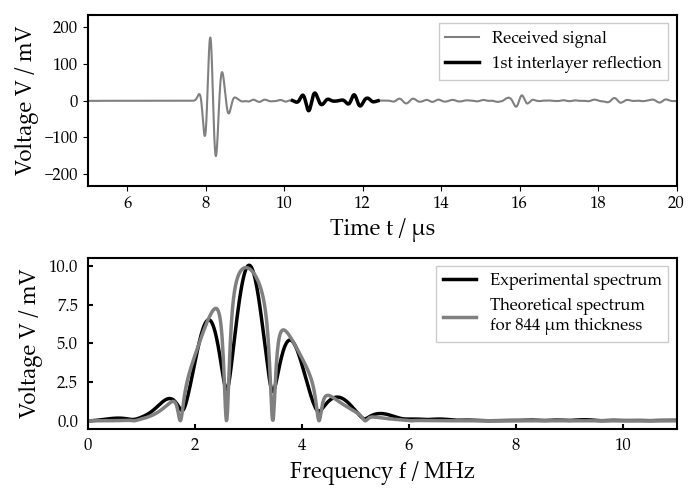

Supplement: Supplementary file 1 [file sensors-23-05942-s001.zip › Figure_S19_Planar_Bone-Water-Titanium-Setup_0900um.png]

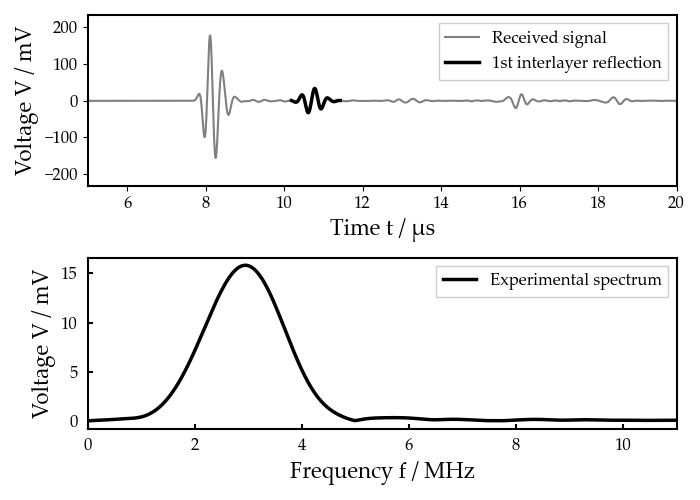

Supplement: Supplementary file 1 [file sensors-23-05942-s001.zip › Figure_S2_Planar_Bone-Water-Titanium-Setup_0050um.png]

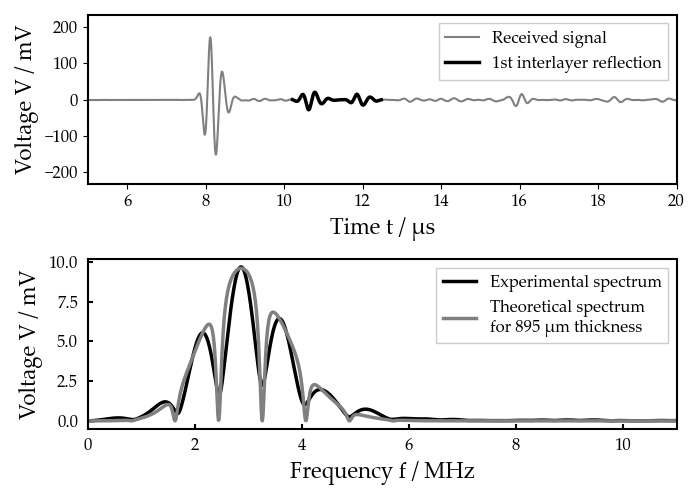

Supplement: Supplementary file 1 [file sensors-23-05942-s001.zip › Figure_S20_Planar_Bone-Water-Titanium-Setup_0950um.png]

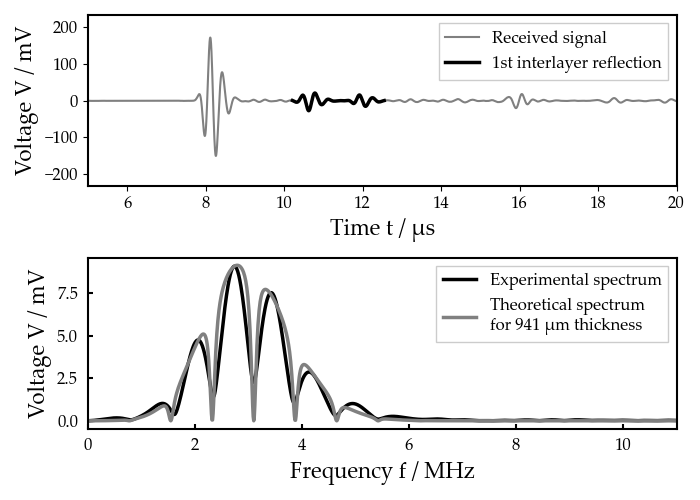

Supplement: Supplementary file 1 [file sensors-23-05942-s001.zip › Figure_S21_Planar_Bone-Water-Titanium-Setup_1000um.png]

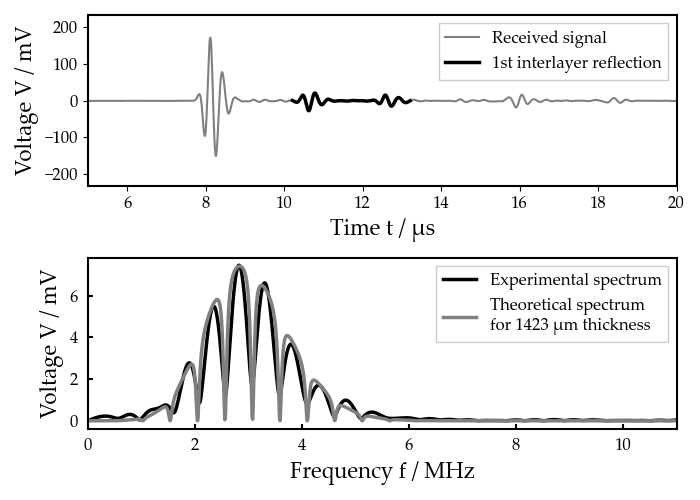

Supplement: Supplementary file 1 [file sensors-23-05942-s001.zip › Figure_S22_Planar_Bone-Water-Titanium-Setup_1500um.png]

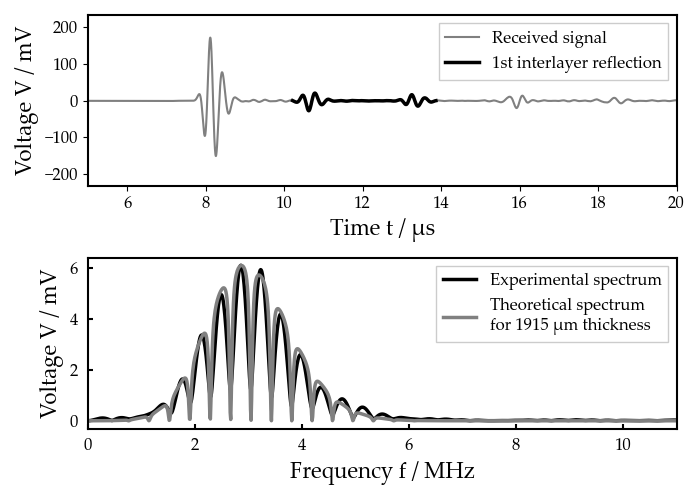

Supplement: Supplementary file 1 [file sensors-23-05942-s001.zip › Figure_S23_Planar_Bone-Water-Titanium-Setup_2000um.png]

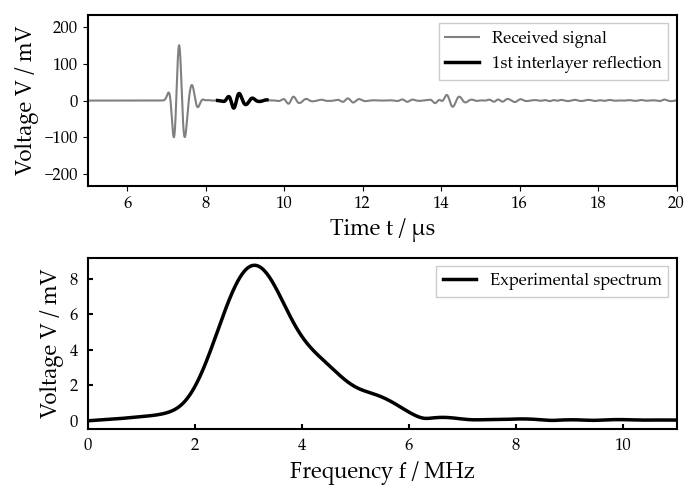

Supplement: Supplementary file 1 [file sensors-23-05942-s001.zip › Figure_S24_Cylindric_Aluminum-Water-Aluminum-Setup_0000um.png]

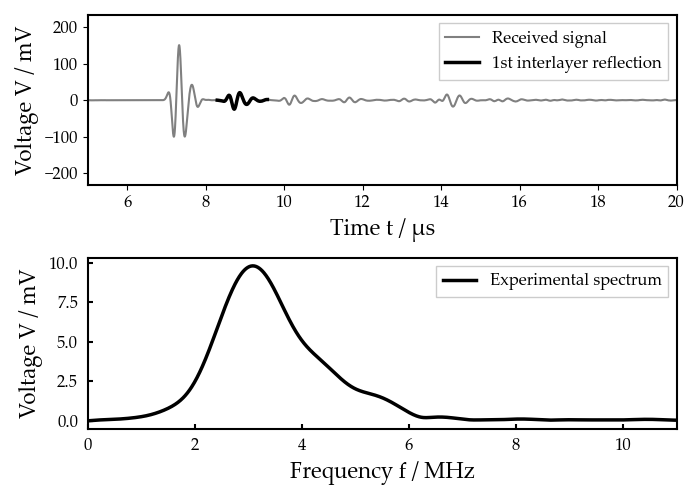

Supplement: Supplementary file 1 [file sensors-23-05942-s001.zip › Figure_S25_Cylindric_Aluminum-Water-Aluminum-Setup_0050um.png]

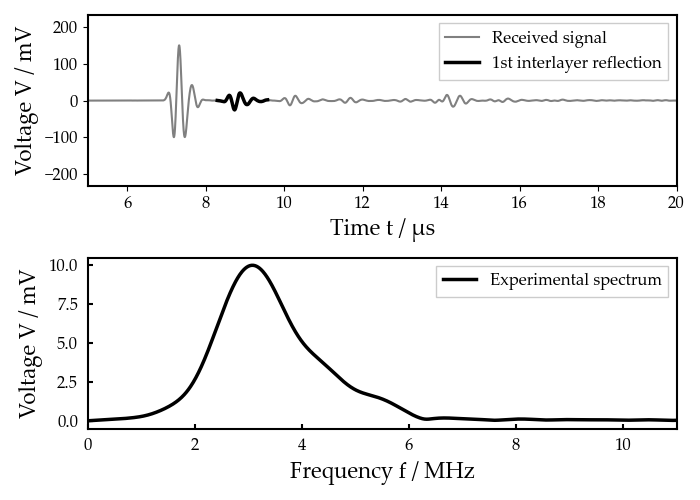

Supplement: Supplementary file 1 [file sensors-23-05942-s001.zip › Figure_S26_Cylindric_Aluminum-Water-Aluminum-Setup_0100um.png]

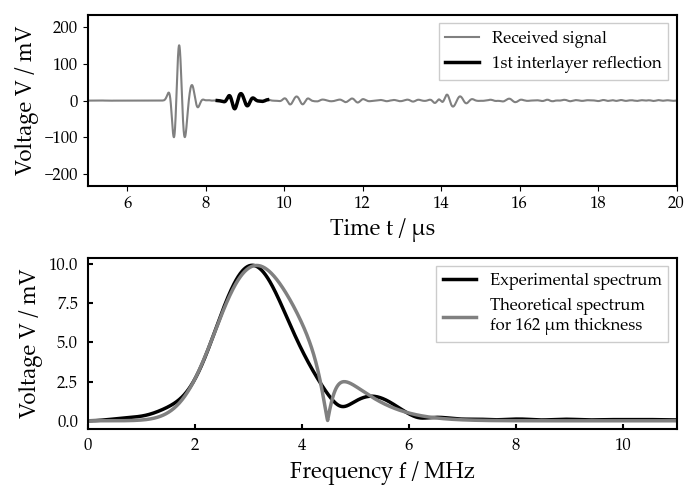

Supplement: Supplementary file 1 [file sensors-23-05942-s001.zip › Figure_S27_Cylindric_Aluminum-Water-Aluminum-Setup_0150um.png]

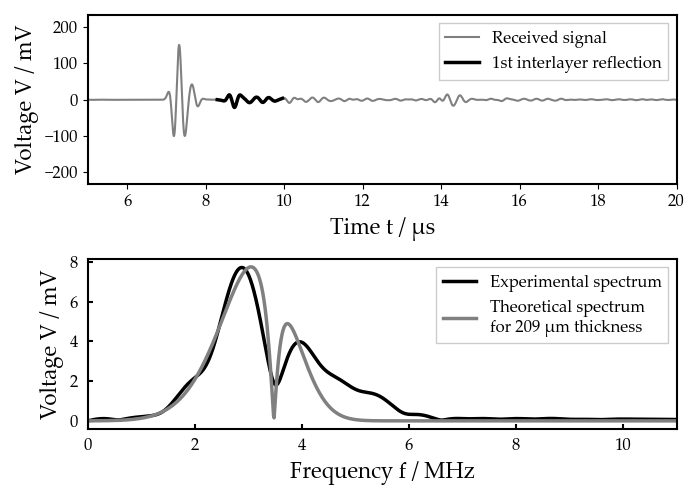

Supplement: Supplementary file 1 [file sensors-23-05942-s001.zip › Figure_S28_Cylindric_Aluminum-Water-Aluminum-Setup_0200um.png]

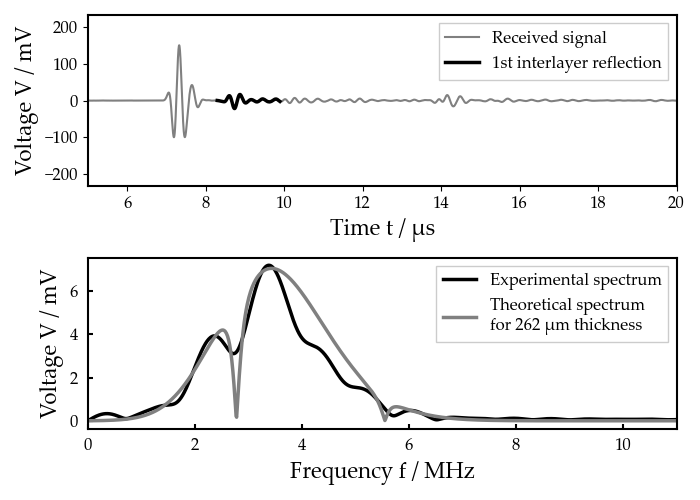

Supplement: Supplementary file 1 [file sensors-23-05942-s001.zip › Figure_S29_Cylindric_Aluminum-Water-Aluminum-Setup_0250um.png]

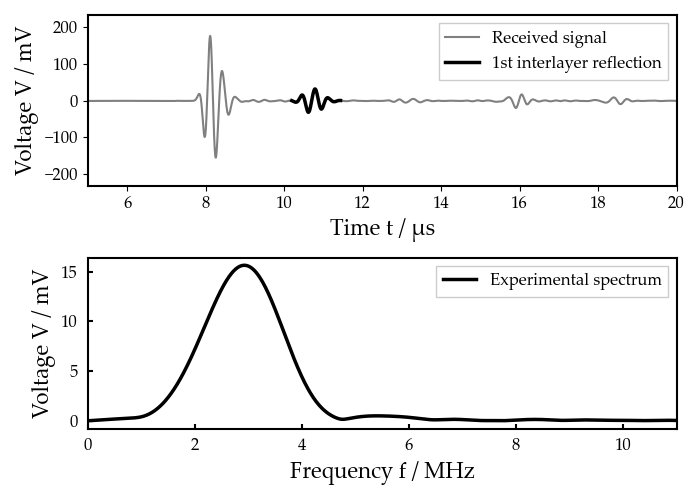

Supplement: Supplementary file 1 [file sensors-23-05942-s001.zip › Figure_S3_Planar_Bone-Water-Titanium-Setup_0100um.png]

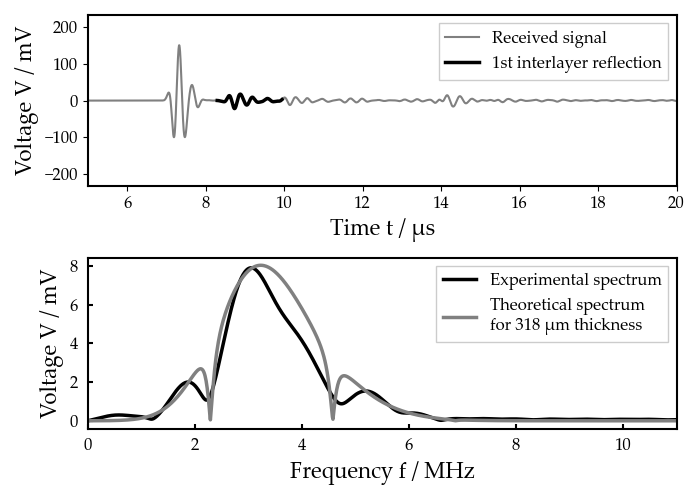

Supplement: Supplementary file 1 [file sensors-23-05942-s001.zip › Figure_S30_Cylindric_Aluminum-Water-Aluminum-Setup_0300um.png]

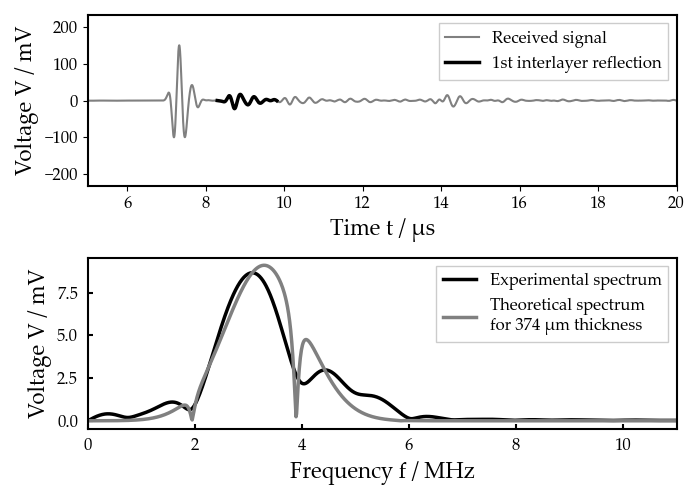

Supplement: Supplementary file 1 [file sensors-23-05942-s001.zip › Figure_S31_Cylindric_Aluminum-Water-Aluminum-Setup_0350um.png]

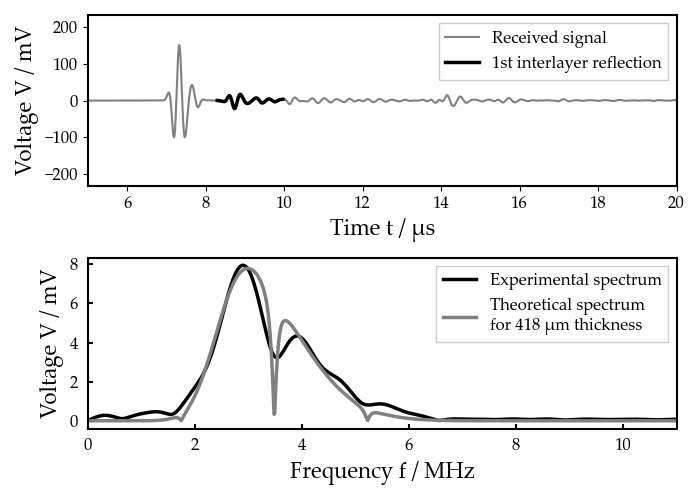

Supplement: Supplementary file 1 [file sensors-23-05942-s001.zip › Figure_S32_Cylindric_Aluminum-Water-Aluminum-Setup_0400um.png]

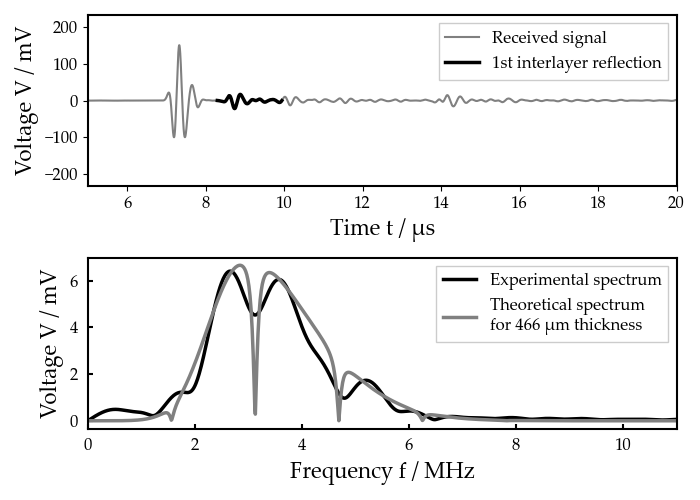

Supplement: Supplementary file 1 [file sensors-23-05942-s001.zip › Figure_S33_Cylindric_Aluminum-Water-Aluminum-Setup_0450um.png]

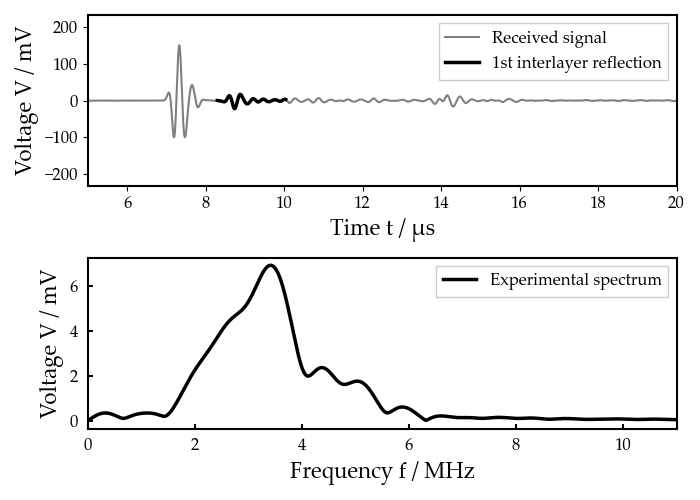

Supplement: Supplementary file 1 [file sensors-23-05942-s001.zip › Figure_S34_Cylindric_Aluminum-Water-Aluminum-Setup_0500um.png]

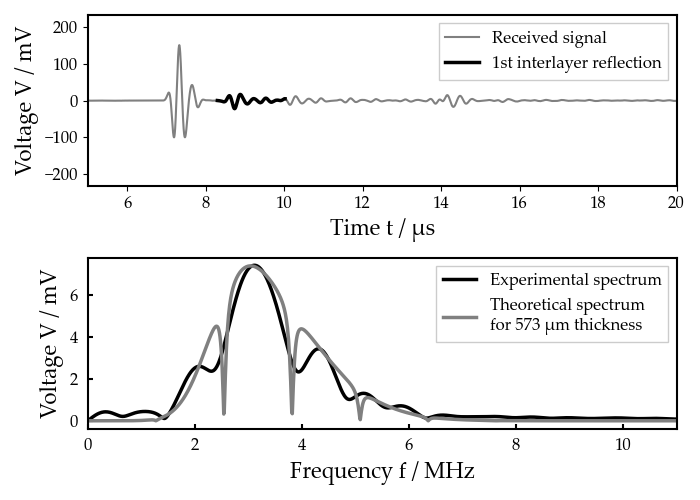

Supplement: Supplementary file 1 [file sensors-23-05942-s001.zip › Figure_S35_Cylindric_Aluminum-Water-Aluminum-Setup_0550um.png]

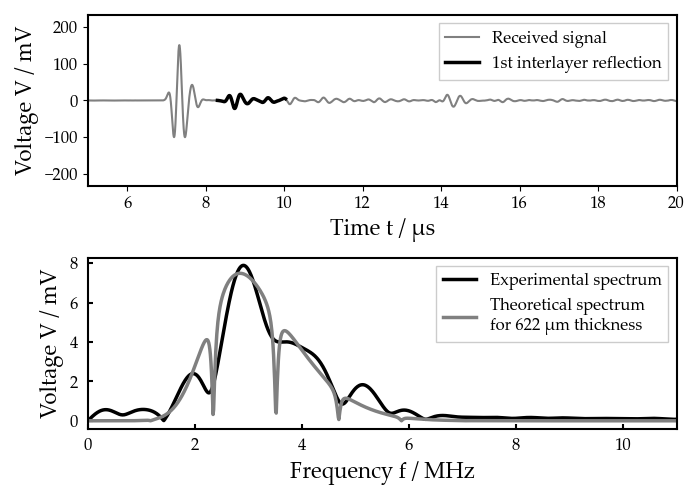

Supplement: Supplementary file 1 [file sensors-23-05942-s001.zip › Figure_S36_Cylindric_Aluminum-Water-Aluminum-Setup_0600um.png]

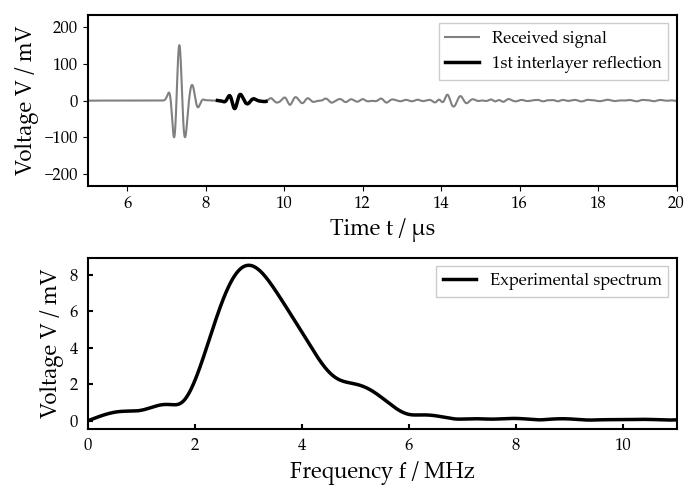

Supplement: Supplementary file 1 [file sensors-23-05942-s001.zip › Figure_S37_Cylindric_Aluminum-Water-Aluminum-Setup_0650um.png]

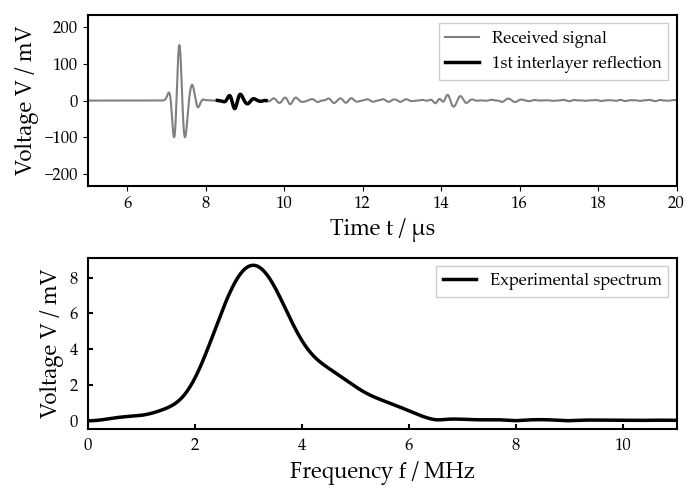

Supplement: Supplementary file 1 [file sensors-23-05942-s001.zip › Figure_S38_Cylindric_Aluminum-Water-Aluminum-Setup_0700um.png]

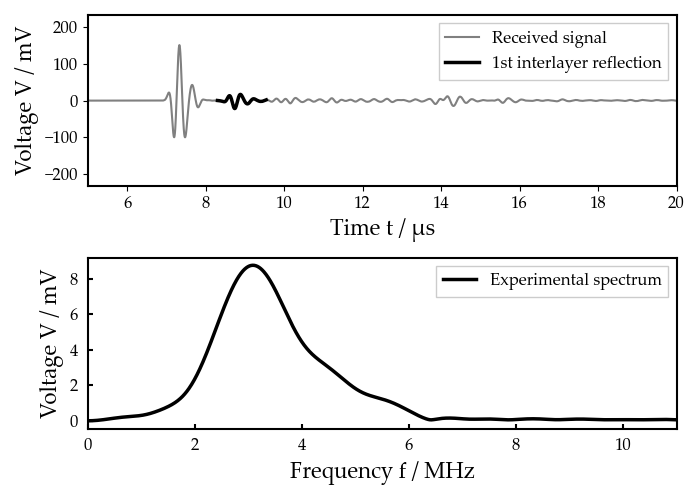

Supplement: Supplementary file 1 [file sensors-23-05942-s001.zip › Figure_S39_Cylindric_Aluminum-Water-Aluminum-Setup_0750um.png]

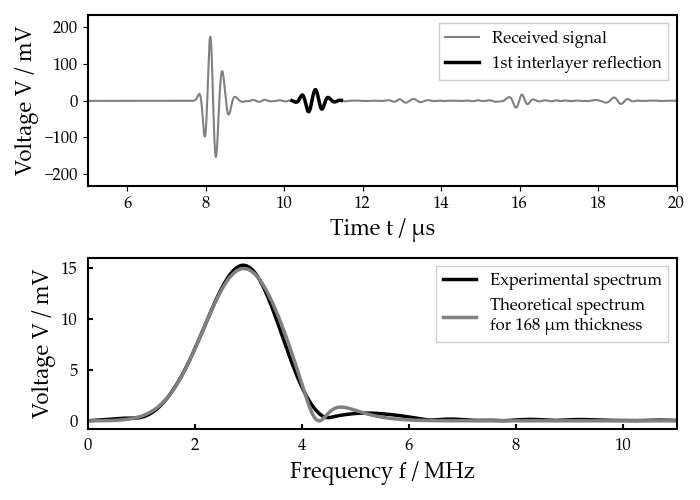

Supplement: Supplementary file 1 [file sensors-23-05942-s001.zip › Figure_S4_Planar_Bone-Water-Titanium-Setup_0150um.png]

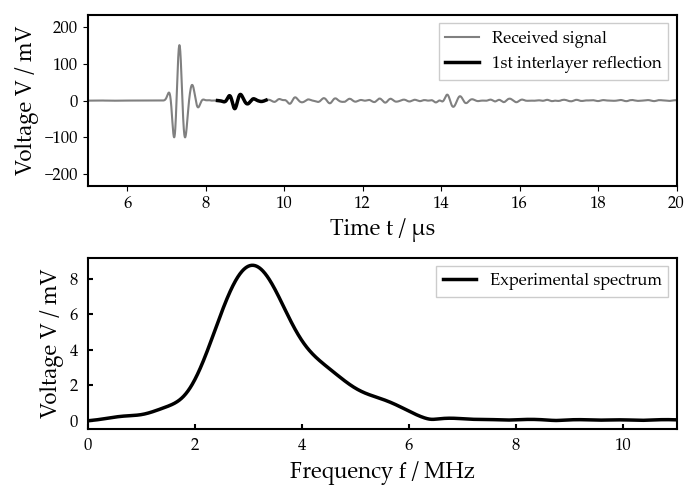

Supplement: Supplementary file 1 [file sensors-23-05942-s001.zip › Figure_S40_Cylindric_Aluminum-Water-Aluminum-Setup_0800um.png]

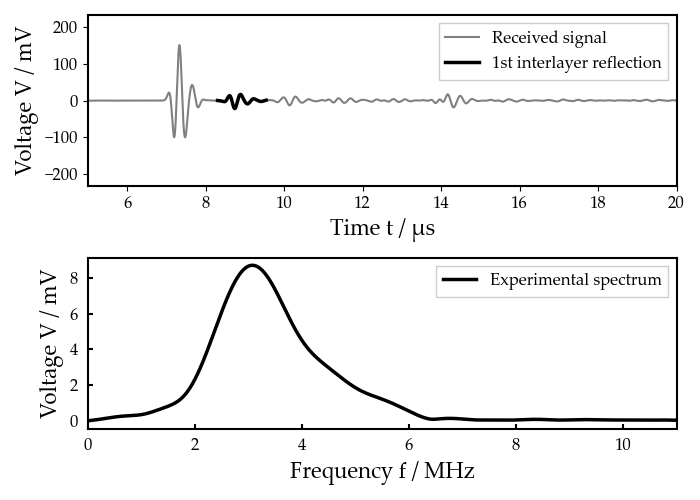

Supplement: Supplementary file 1 [file sensors-23-05942-s001.zip › Figure_S41_Cylindric_Aluminum-Water-Aluminum-Setup_0850um.png]

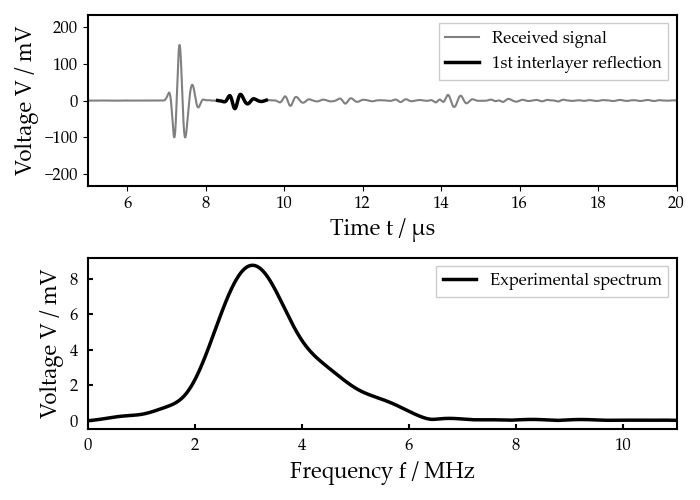

Supplement: Supplementary file 1 [file sensors-23-05942-s001.zip › Figure_S42_Cylindric_Aluminum-Water-Aluminum-Setup_0900um.png]

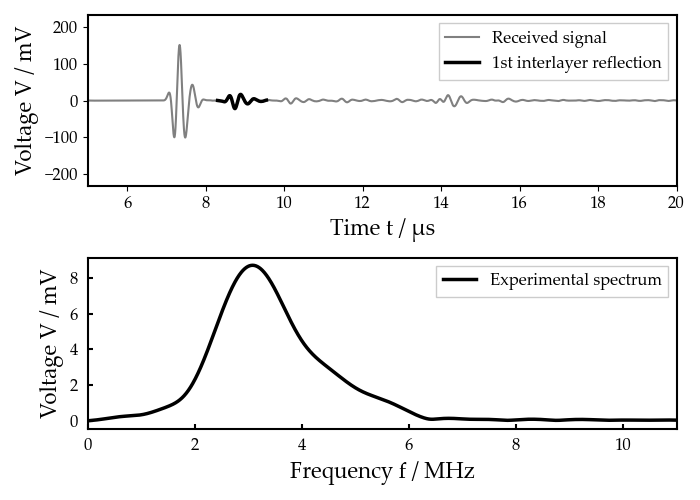

Supplement: Supplementary file 1 [file sensors-23-05942-s001.zip › Figure_S43_Cylindric_Aluminum-Water-Aluminum-Setup_0950um.png]

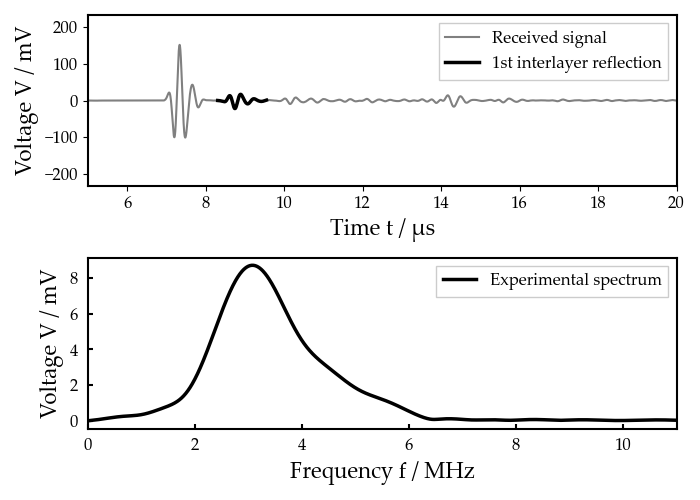

Supplement: Supplementary file 1 [file sensors-23-05942-s001.zip › Figure_S44_Cylindric_Aluminum-Water-Aluminum-Setup_1000um.png]

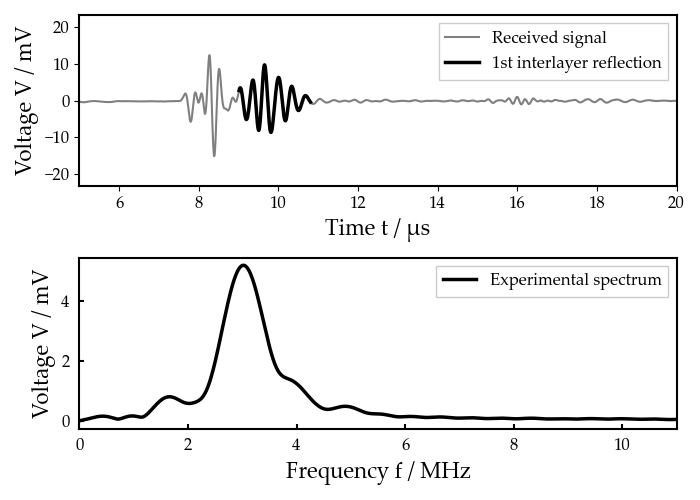

Supplement: Supplementary file 1 [file sensors-23-05942-s001.zip › Figure_S45_Bone-Implant-Setup_S1_M1.png]

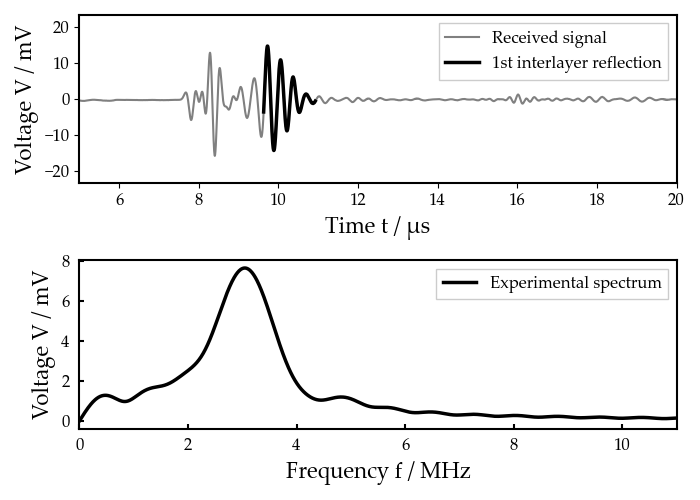

Supplement: Supplementary file 1 [file sensors-23-05942-s001.zip › Figure_S46_Bone-Implant-Setup_S1_M2.png]

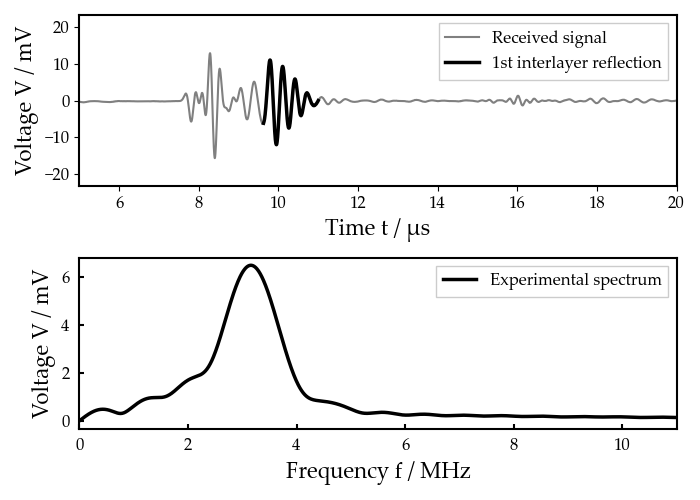

Supplement: Supplementary file 1 [file sensors-23-05942-s001.zip › Figure_S47_Bone-Implant-Setup_S1_M3.png]

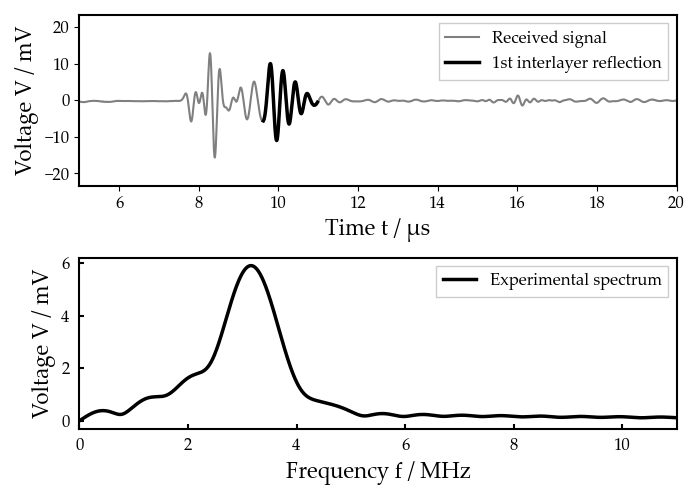

Supplement: Supplementary file 1 [file sensors-23-05942-s001.zip › Figure_S48_Bone-Implant-Setup_S1_M4.png]

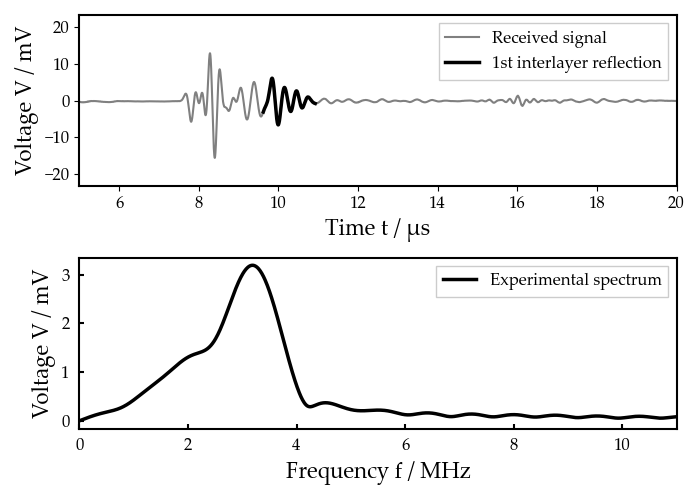

Supplement: Supplementary file 1 [file sensors-23-05942-s001.zip › Figure_S49_Bone-Implant-Setup_S1_M5.png]

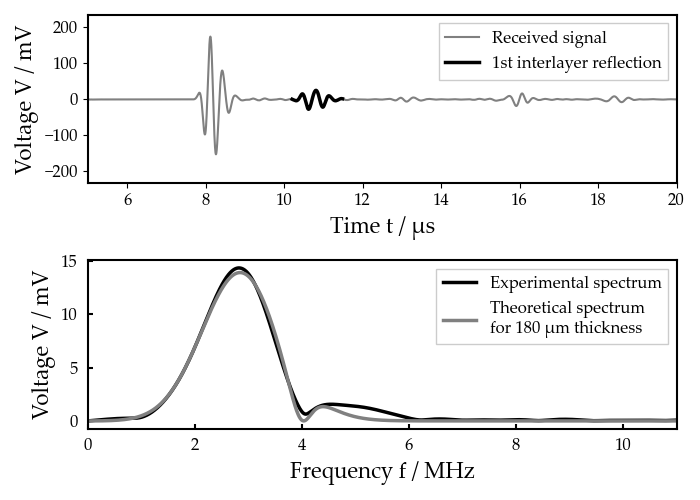

Supplement: Supplementary file 1 [file sensors-23-05942-s001.zip › Figure_S5_Planar_Bone-Water-Titanium-Setup_0200um.png]

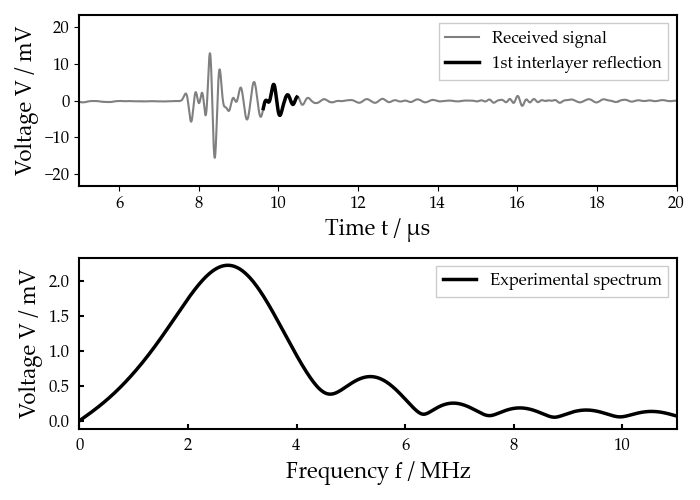

Supplement: Supplementary file 1 [file sensors-23-05942-s001.zip › Figure_S50_Bone-Implant-Setup_S1_M6.png]

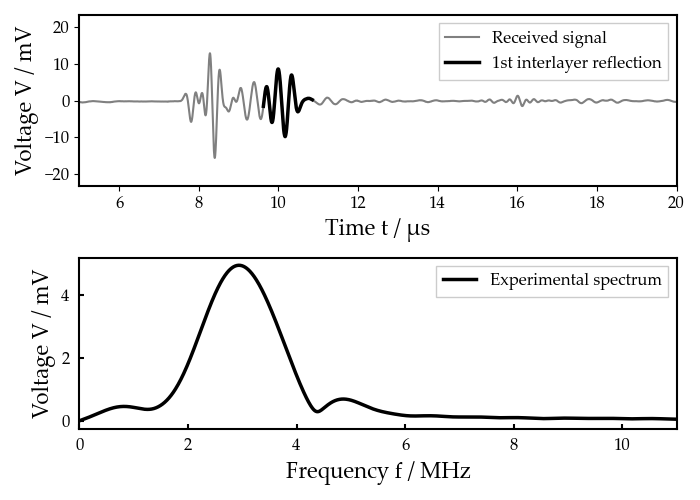

Supplement: Supplementary file 1 [file sensors-23-05942-s001.zip › Figure_S51_Bone-Implant-Setup_S1_M7.png]

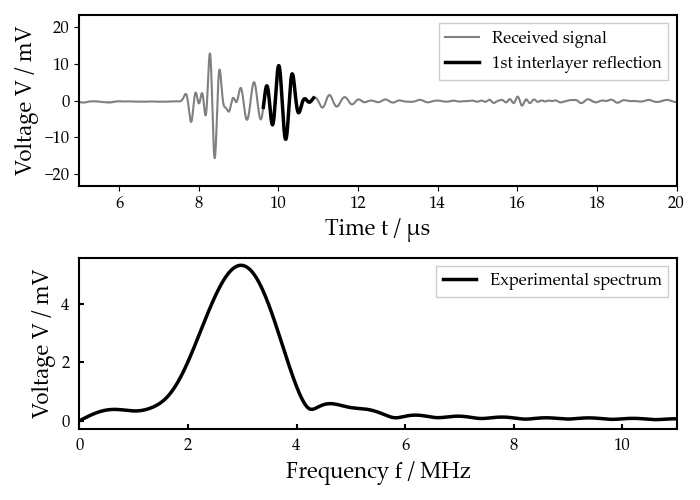

Supplement: Supplementary file 1 [file sensors-23-05942-s001.zip › Figure_S52_Bone-Implant-Setup_S1_M8.png]

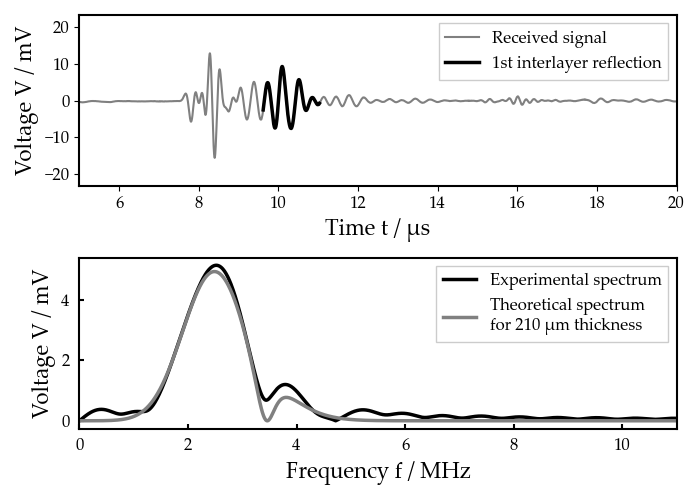

Supplement: Supplementary file 1 [file sensors-23-05942-s001.zip › Figure_S53_Bone-Implant-Setup_S1_M9.png]

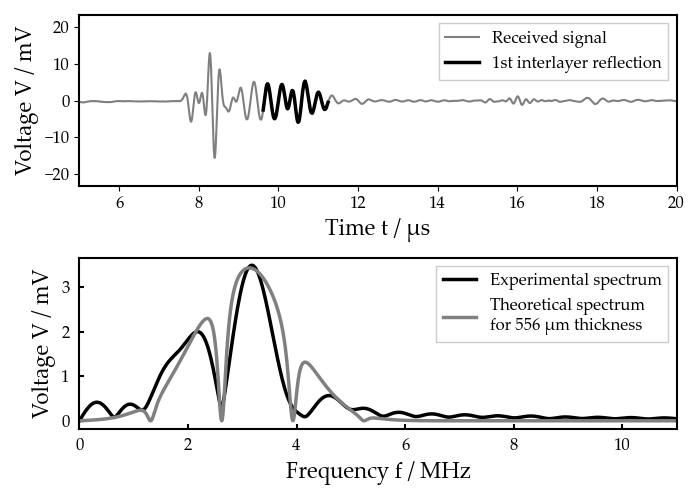

Supplement: Supplementary file 1 [file sensors-23-05942-s001.zip › Figure_S54_Bone-Implant-Setup_S1_M10.png]

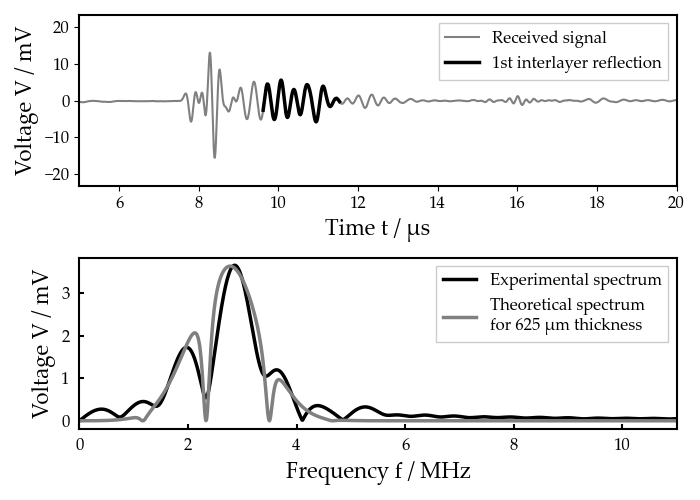

Supplement: Supplementary file 1 [file sensors-23-05942-s001.zip › Figure_S55_Bone-Implant-Setup_S1_M11.png]

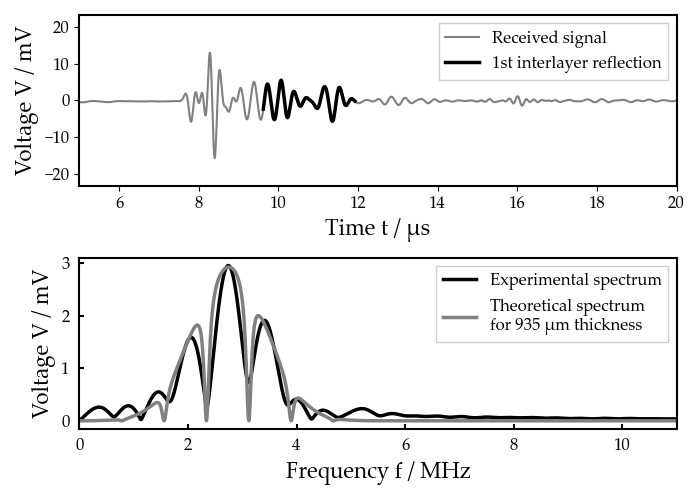

Supplement: Supplementary file 1 [file sensors-23-05942-s001.zip › Figure_S56_Bone-Implant-Setup_S1_M12.png]

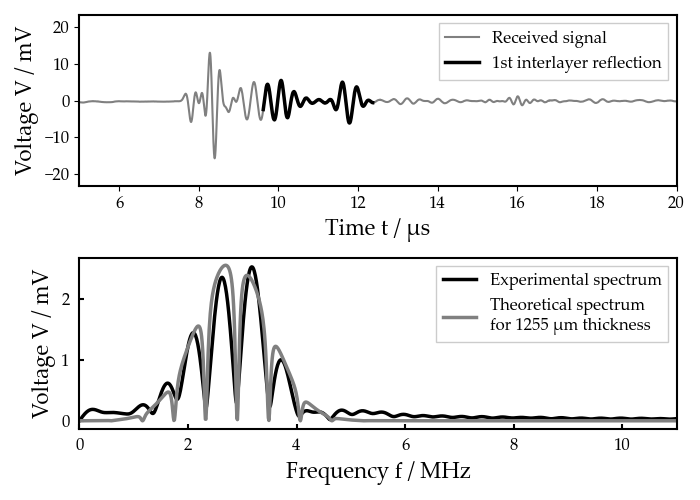

Supplement: Supplementary file 1 [file sensors-23-05942-s001.zip › Figure_S57_Bone-Implant-Setup_S1_M13.png]

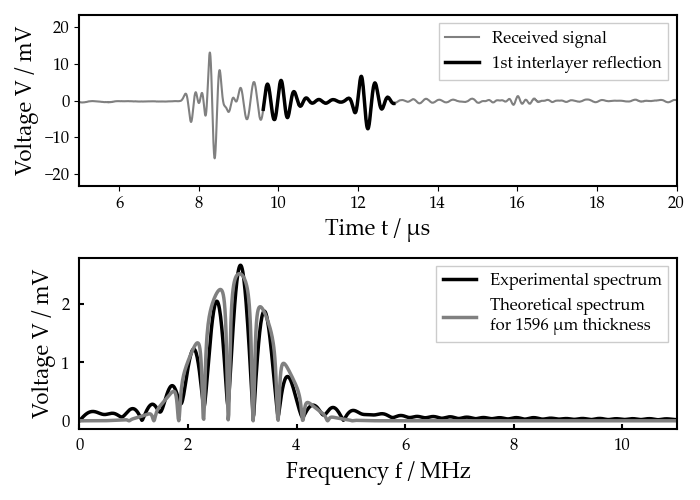

Supplement: Supplementary file 1 [file sensors-23-05942-s001.zip › Figure_S58_Bone-Implant-Setup_S1_M14.png]

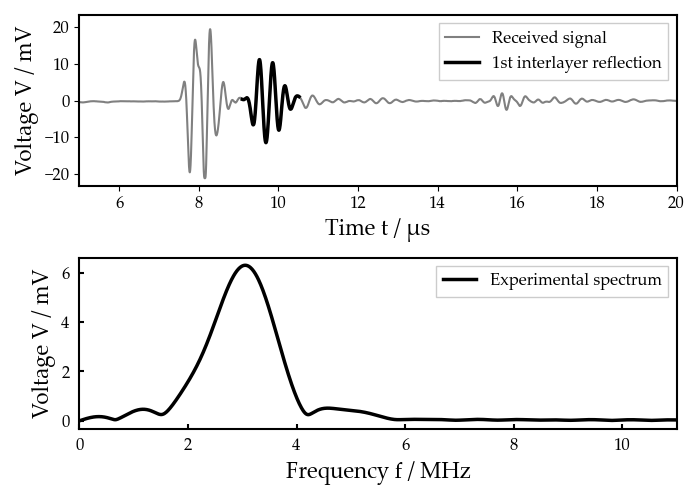

Supplement: Supplementary file 1 [file sensors-23-05942-s001.zip › Figure_S59_Bone-Implant-Setup_S2_M1.png]

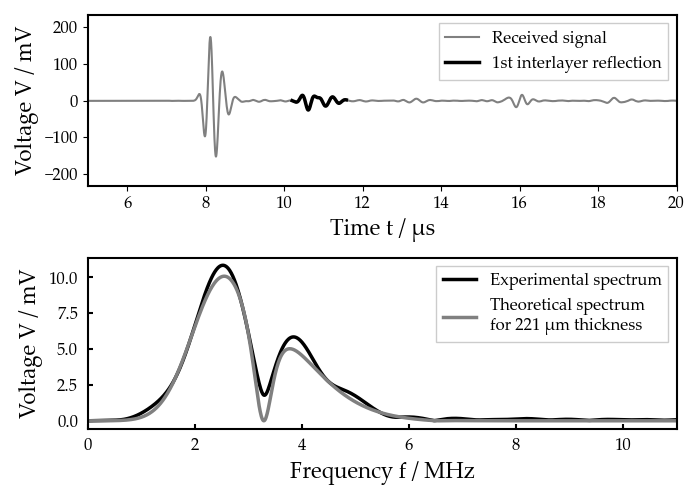

Supplement: Supplementary file 1 [file sensors-23-05942-s001.zip › Figure_S6_Planar_Bone-Water-Titanium-Setup_0250um.png]

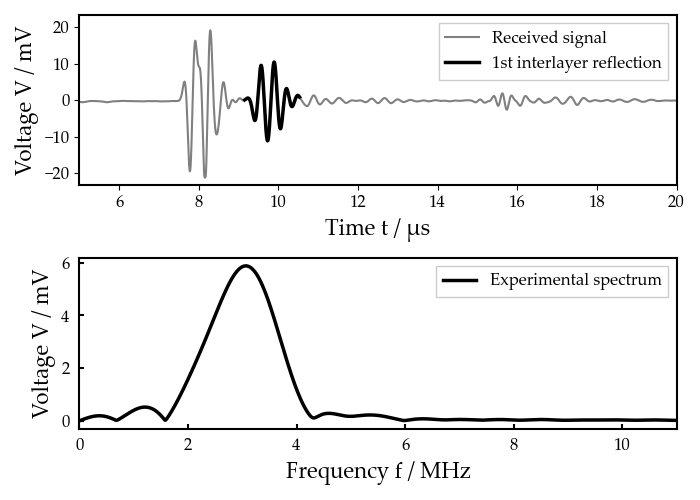

Supplement: Supplementary file 1 [file sensors-23-05942-s001.zip › Figure_S60_Bone-Implant-Setup_S2_M2.png]

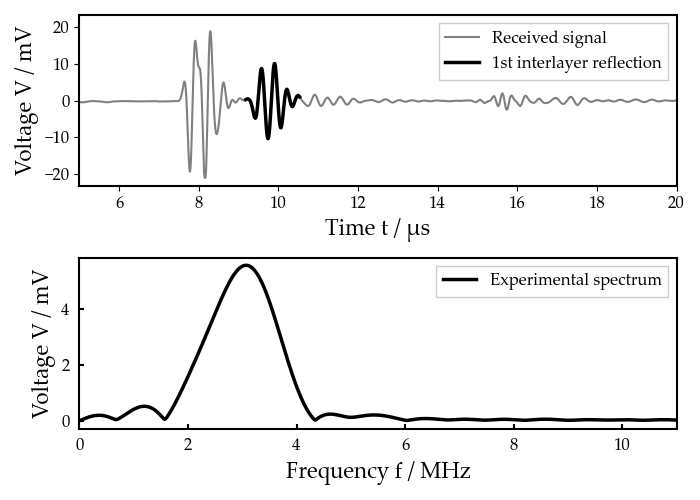

Supplement: Supplementary file 1 [file sensors-23-05942-s001.zip › Figure_S61_Bone-Implant-Setup_S2_M3.png]

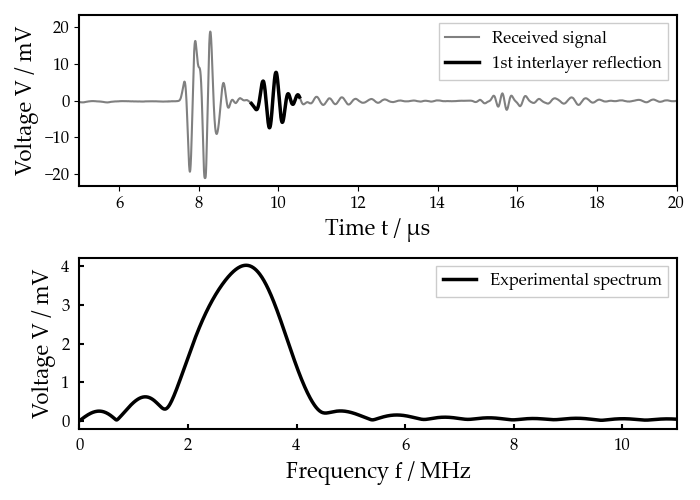

Supplement: Supplementary file 1 [file sensors-23-05942-s001.zip › Figure_S62_Bone-Implant-Setup_S2_M4.png]

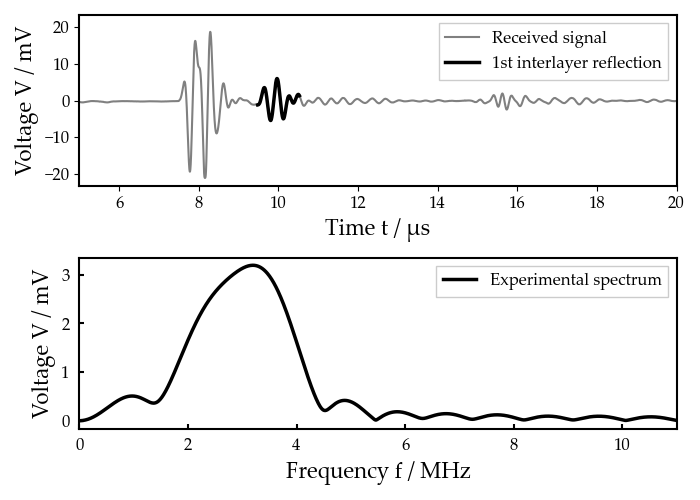

Supplement: Supplementary file 1 [file sensors-23-05942-s001.zip › Figure_S63_Bone-Implant-Setup_S2_M5.png]

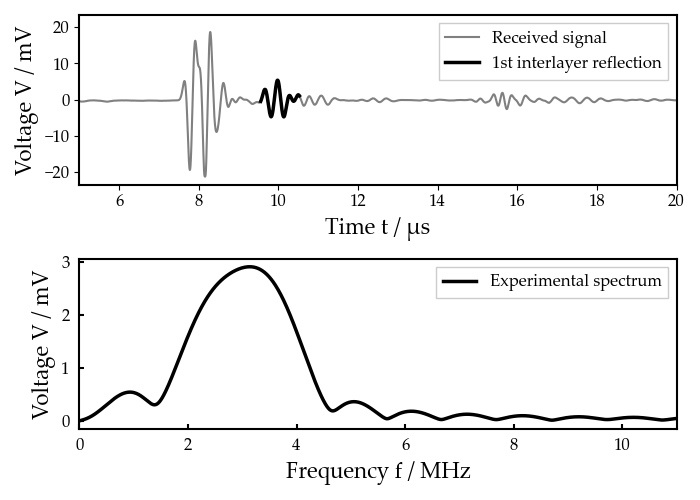

Supplement: Supplementary file 1 [file sensors-23-05942-s001.zip › Figure_S64_Bone-Implant-Setup_S2_M6.png]

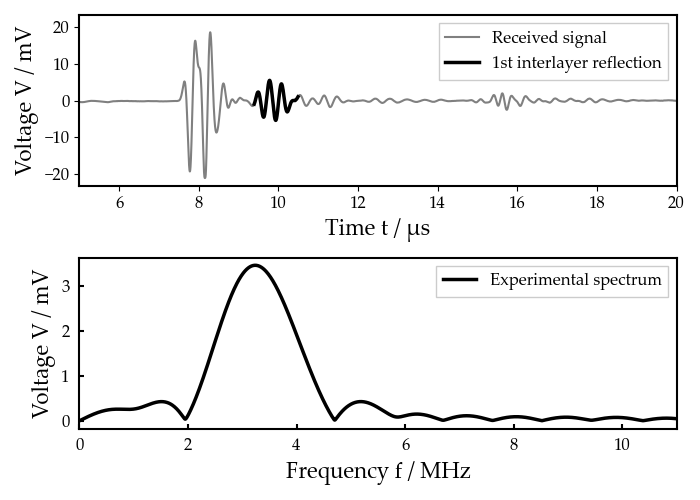

Supplement: Supplementary file 1 [file sensors-23-05942-s001.zip › Figure_S65_Bone-Implant-Setup_S2_M7.png]

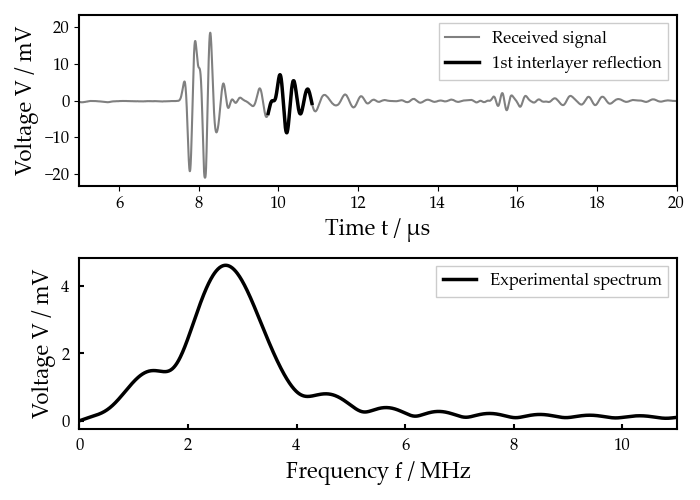

Supplement: Supplementary file 1 [file sensors-23-05942-s001.zip › Figure_S66_Bone-Implant-Setup_S2_M8.png]

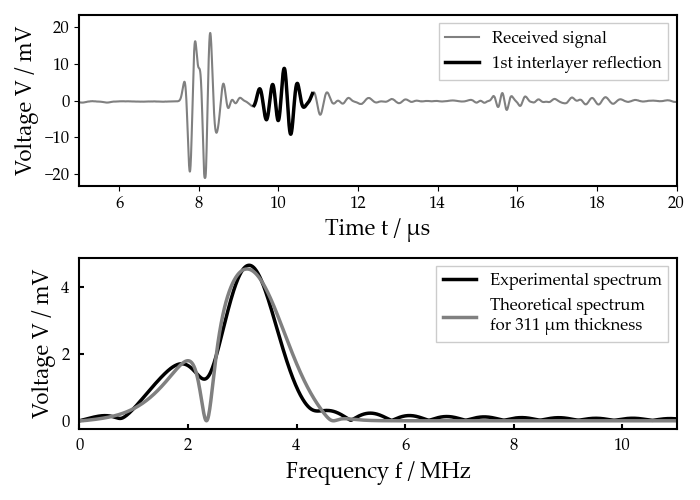

Supplement: Supplementary file 1 [file sensors-23-05942-s001.zip › Figure_S67_Bone-Implant-Setup_S2_M9.png]

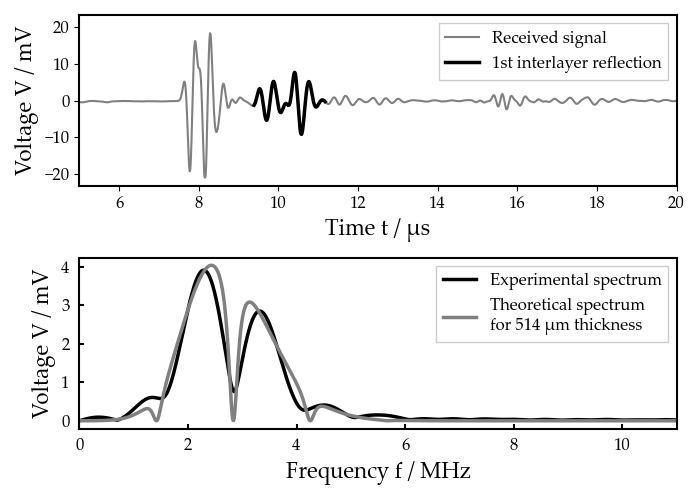

Supplement: Supplementary file 1 [file sensors-23-05942-s001.zip › Figure_S68_Bone-Implant-Setup_S2_M11.png]

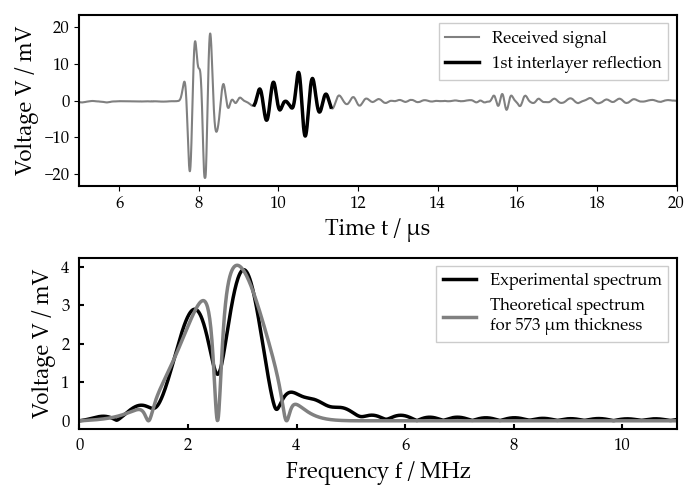

Supplement: Supplementary file 1 [file sensors-23-05942-s001.zip › Figure_S69_Bone-Implant-Setup_S2_M12.png]

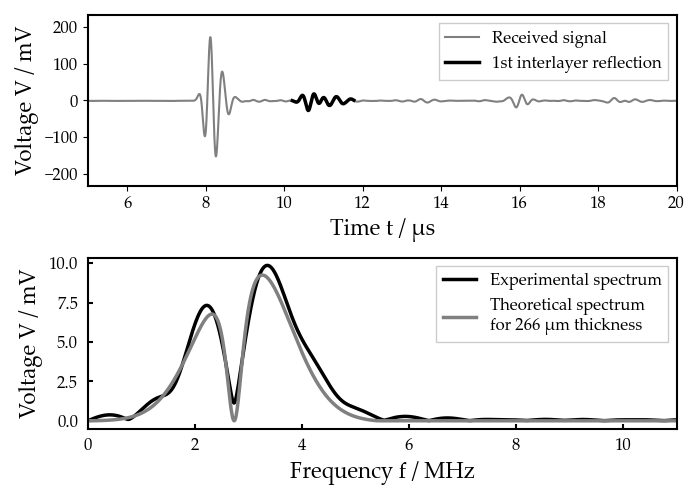

Supplement: Supplementary file 1 [file sensors-23-05942-s001.zip › Figure_S7_Planar_Bone-Water-Titanium-Setup_0300um.png]

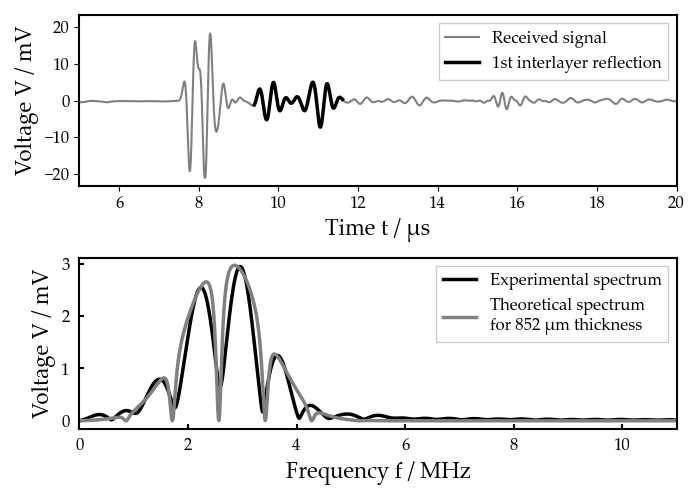

Supplement: Supplementary file 1 [file sensors-23-05942-s001.zip › Figure_S70_Bone-Implant-Setup_S2_M13.png]

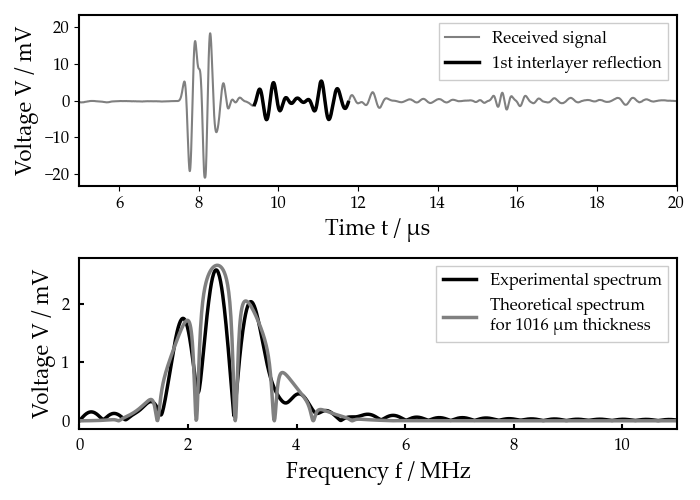

Supplement: Supplementary file 1 [file sensors-23-05942-s001.zip › Figure_S71_Bone-Implant-Setup_S2_M14.png]

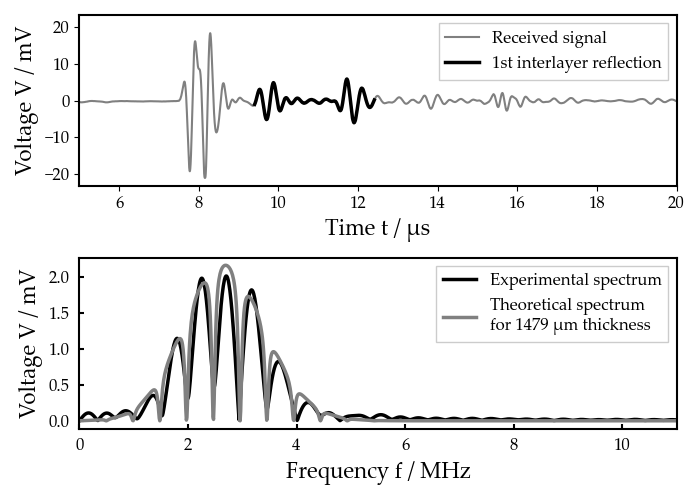

Supplement: Supplementary file 1 [file sensors-23-05942-s001.zip › Figure_S72_Bone-Implant-Setup_S2_M15.png]

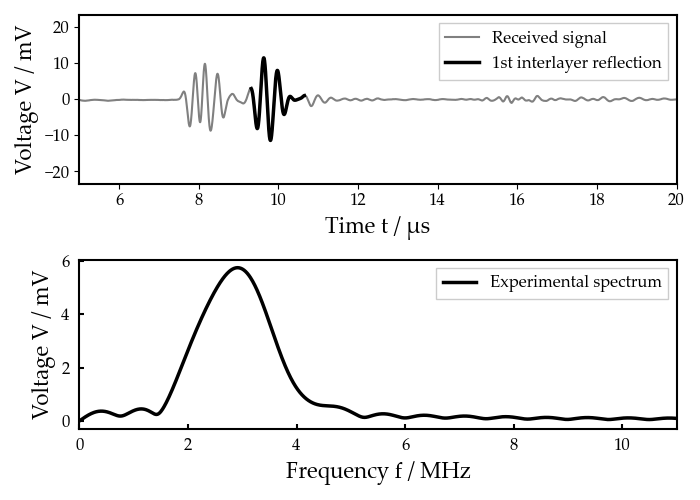

Supplement: Supplementary file 1 [file sensors-23-05942-s001.zip › Figure_S73_Bone-Implant-Setup_S3_M1.png]

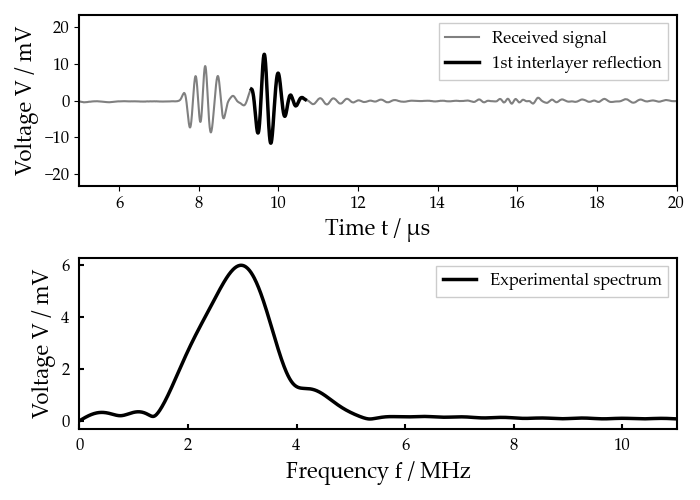

Supplement: Supplementary file 1 [file sensors-23-05942-s001.zip › Figure_S74_Bone-Implant-Setup_S3_M2.png]

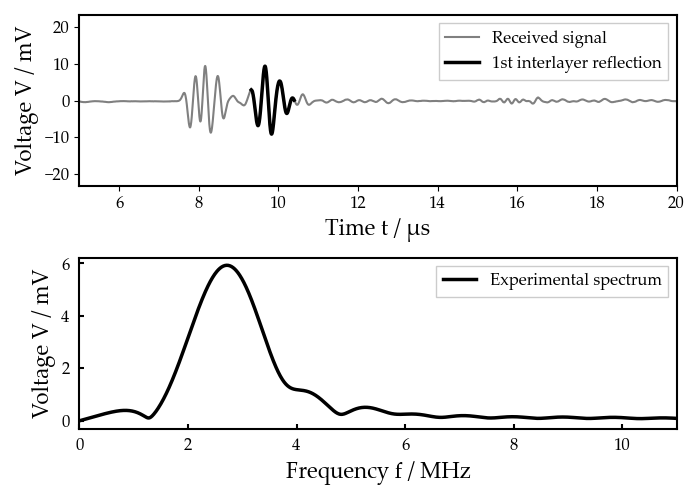

Supplement: Supplementary file 1 [file sensors-23-05942-s001.zip › Figure_S75_Bone-Implant-Setup_S3_M3.png]

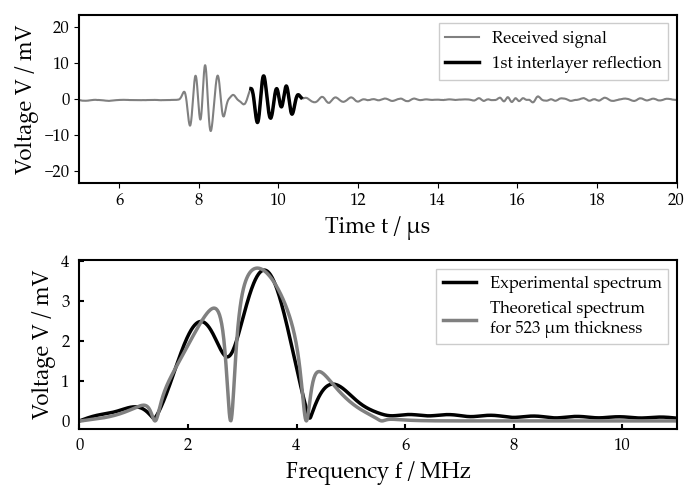

Supplement: Supplementary file 1 [file sensors-23-05942-s001.zip › Figure_S76_Bone-Implant-Setup_S3_M4.png]

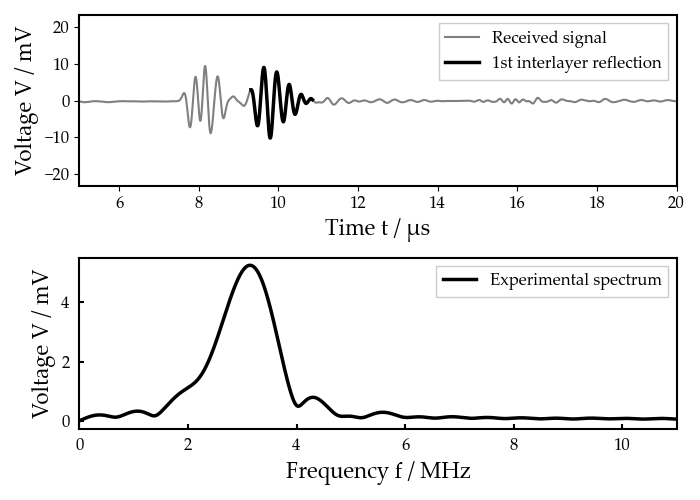

Supplement: Supplementary file 1 [file sensors-23-05942-s001.zip › Figure_S77_Bone-Implant-Setup_S3_M5.png]

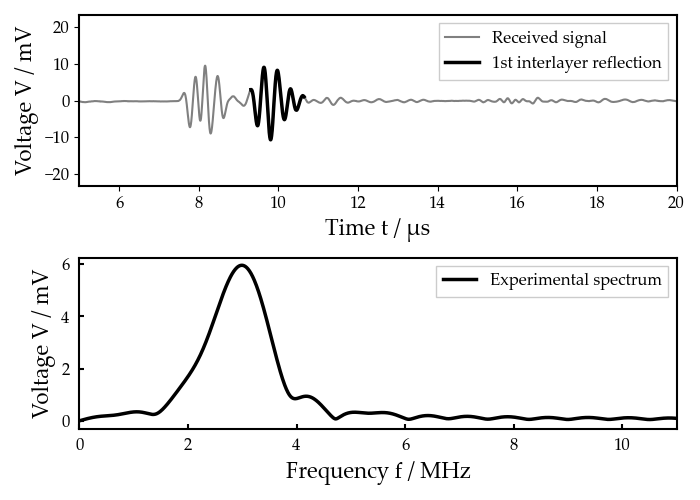

Supplement: Supplementary file 1 [file sensors-23-05942-s001.zip › Figure_S78_Bone-Implant-Setup_S3_M6.png]

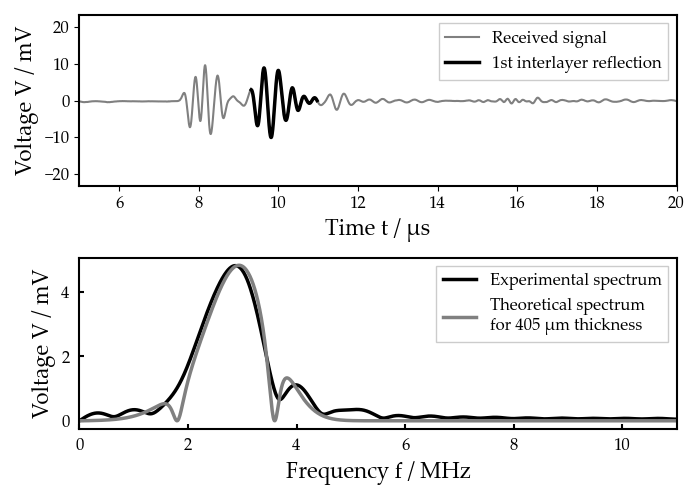

Supplement: Supplementary file 1 [file sensors-23-05942-s001.zip › Figure_S79_Bone-Implant-Setup_S3_M7.png]

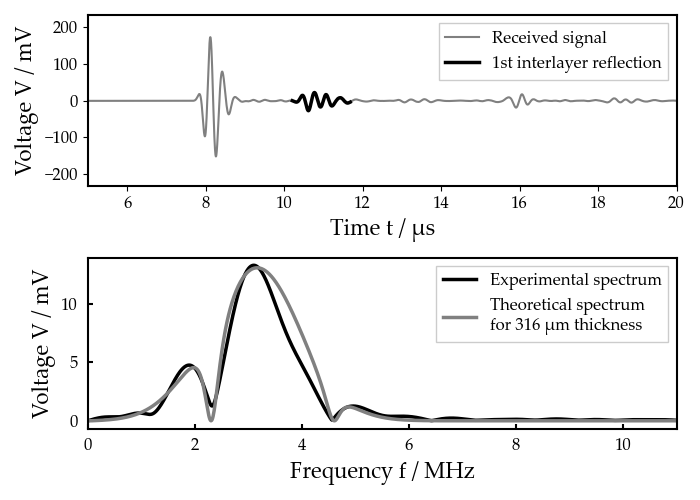

Supplement: Supplementary file 1 [file sensors-23-05942-s001.zip › Figure_S8_Planar_Bone-Water-Titanium-Setup_0350um.png]

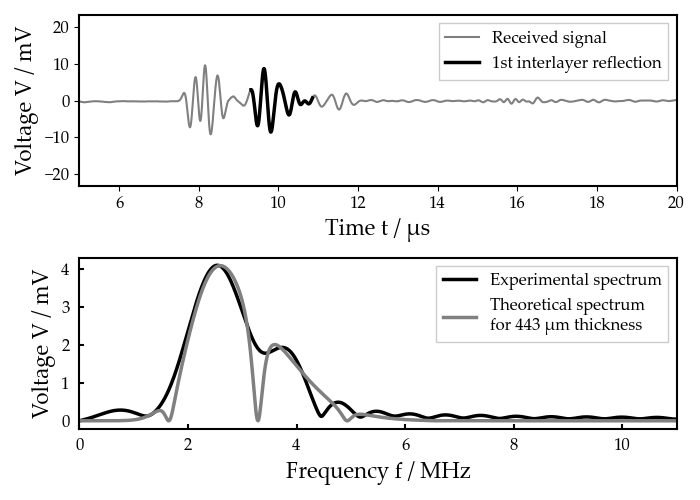

Supplement: Supplementary file 1 [file sensors-23-05942-s001.zip › Figure_S80_Bone-Implant-Setup_S3_M8.png]

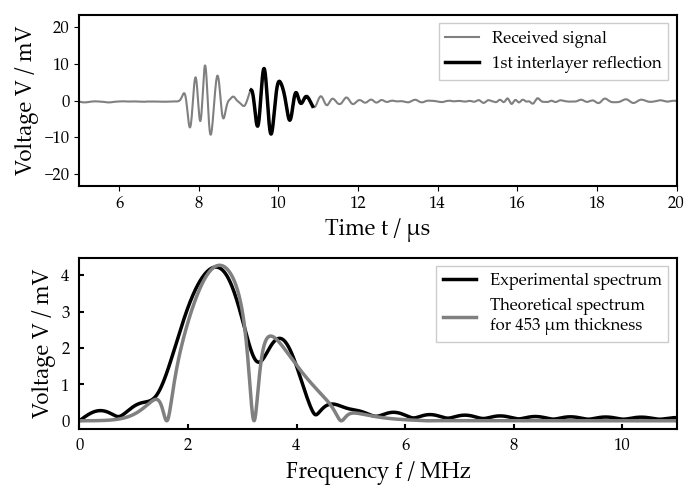

Supplement: Supplementary file 1 [file sensors-23-05942-s001.zip › Figure_S81_Bone-Implant-Setup_S3_M9.png]

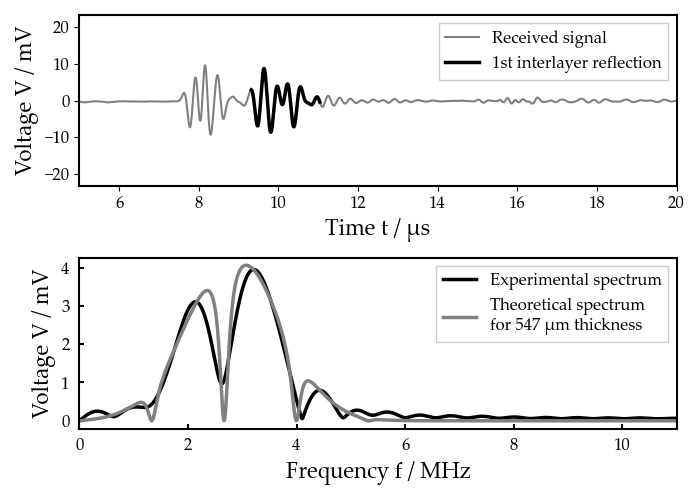

Supplement: Supplementary file 1 [file sensors-23-05942-s001.zip › Figure_S82_Bone-Implant-Setup_S3_M10.png]

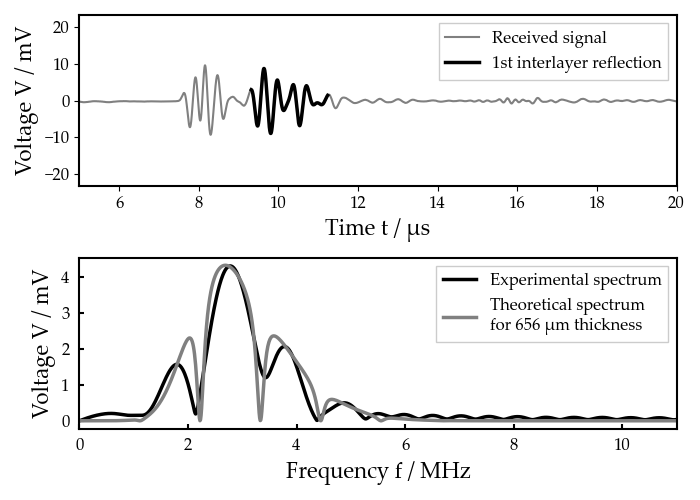

Supplement: Supplementary file 1 [file sensors-23-05942-s001.zip › Figure_S83_Bone-Implant-Setup_S3_M11.png]

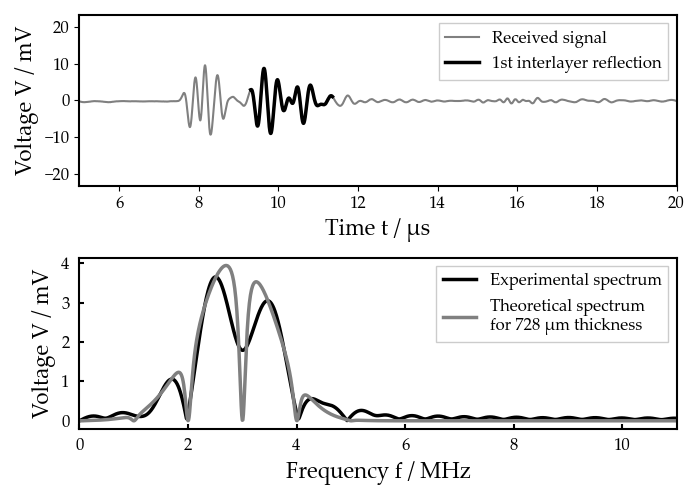

Supplement: Supplementary file 1 [file sensors-23-05942-s001.zip › Figure_S84_Bone-Implant-Setup_S3_M12.png]

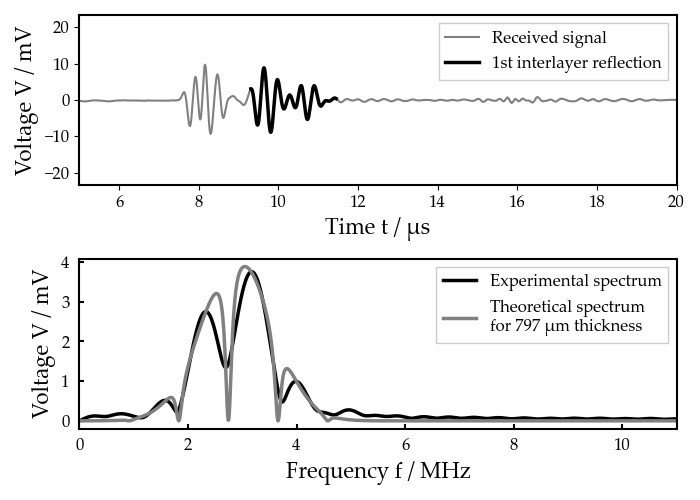

Supplement: Supplementary file 1 [file sensors-23-05942-s001.zip › Figure_S85_Bone-Implant-Setup_S3_M13.png]

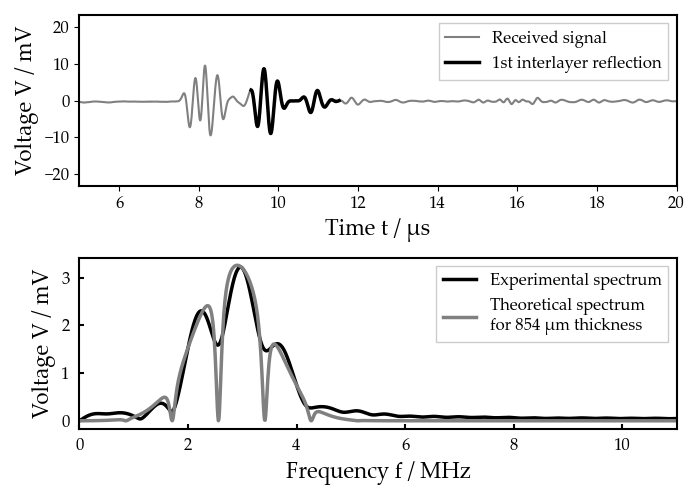

Supplement: Supplementary file 1 [file sensors-23-05942-s001.zip › Figure_S86_Bone-Implant-Setup_S3_M14.png]

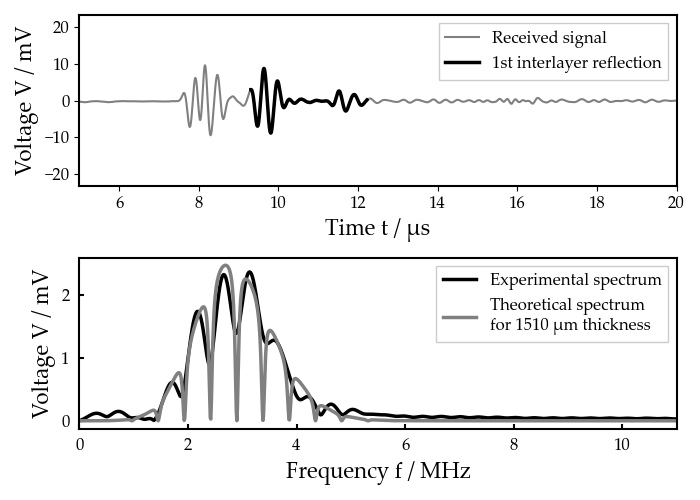

Supplement: Supplementary file 1 [file sensors-23-05942-s001.zip › Figure_S87_Bone-Implant-Setup_S3_M15.png]

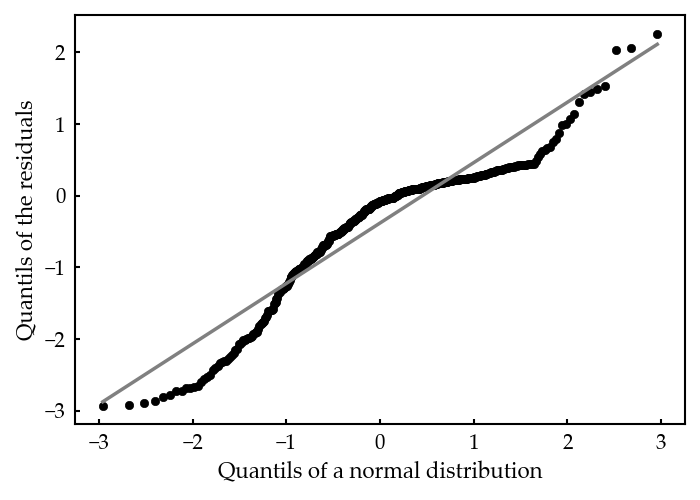

Supplement: Supplementary file 1 [file sensors-23-05942-s001.zip › Figure_S88_QQ-Plot_Residuals_Planar_Bone-Water-Titanium-Setup_1000um.png]

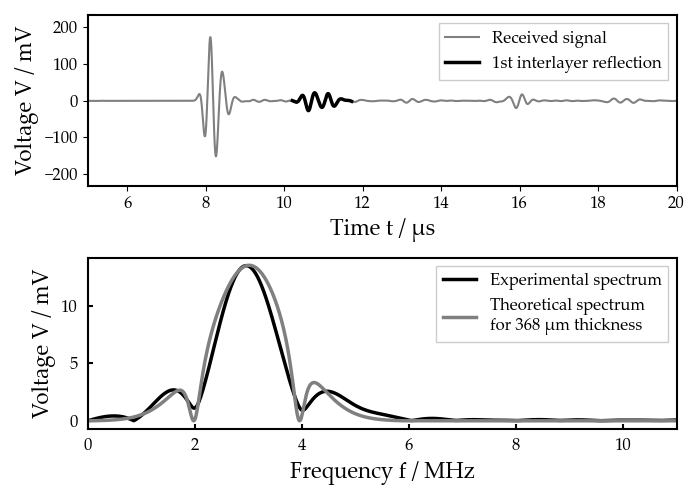

Supplement: Supplementary file 1 [file sensors-23-05942-s001.zip › Figure_S9_Planar_Bone-Water-Titanium-Setup_0400um.png]
